# Internet-Based Brief Personalized Feedback Intervention in a Non-Treatment-Seeking Population of Adult Heavy Drinkers: A Randomized Controlled Trial

**DOI:** 10.2196/jmir.1883

**Published:** 2012-07-30

**Authors:** Anders Blædel Gottlieb Hansen, Ulrik Becker, Anette Søgaard Nielsen, Morten Grønbæk, Janne Schurmann Tolstrup, Lau Caspar Thygesen

**Affiliations:** ^1^National Institute of Public HealthFaculty of Health SciencesUniversity of Southern DenmarkCopenhagenDenmark; ^2^Department of Medical GastroenterologyCopenhagen University HospitalCapital RegionHvidovreDenmark; ^3^The TreatmentcenterOdenseDenmark

**Keywords:** Internet-based personalized feedback, normative feedback, alcohol, heavy drinking, adult, Internet-based personalized brief advice, brief intervention

## Abstract

**Background:**

Internet-based interventions for heavy drinkers show promising results, but existing research is characterized by few studies in nonstudent adult populations and few comparisons with appropriate control groups.

**Objective:**

To test whether a fully automated Internet-based brief personalized feedback intervention and a fully automated Internet-based personalized brief advice intervention in a non-treatment-seeking population of heavy drinkers would result in a reduced alcohol intake.

**Methods:**

We conducted a 3-arm parallel randomized controlled trial in a general population-based sample of heavy drinkers. The 54,157 participants (median age of 58 years) were screened for heavy drinking. Of the 3418 participants who had a weekly alcohol consumption above 14 drinks for women and 21 drinks for men, 1380 (619 women) consented to take part in the trial and were randomly assigned to an Internet-based brief personalized feedback intervention group (normative feedback, n = 476), an Internet-based personalized brief advice intervention group (n = 450), or a nonintervention control group (n = 454). Follow-up after 6 and 12 months included 871 and 1064 participants, respectively, of all groups combined. The outcome measure was self-reported weekly alcohol consumption. We analyzed the data according to the intention-to-treat principle. To examine changes over time and to account for the multiple time measurements, we used a multilevel linear mixed model. To take attrition into account, we used multiple imputation to address missing data.

**Results:**

The intervention effect of the Internet-based brief personalized feedback intervention, determined as the mean additional difference in changes in alcohol consumption in the Internet-based brief personalized feedback intervention compared with the control group, was –1.8 drinks/week after 6 months and –1.4 drinks/week after 12 months; these effects were nonsignificant (95% confidence interval –4.0 to 0.3 at 6 months, –3.4 to 0.6 at 12 months). The intervention effect of the Internet-based personalized brief advice intervention was –0.5 drinks/week after 6 months and –1.2 drinks/week after 12 months; these effects were nonsignificant (95% confidence interval –2.7 to 1.6 at 6 months, –3.3 to 0.9 at 12 months).

**Conclusions:**

In this randomized controlled trial we found no evidence that an Internet-based brief personalized feedback intervention was effective in reducing drinking in an adult population of heavy drinkers.

**Trial registration:**

ClinicalTrials.gov NCT00751985; http://clinicaltrials.gov/ct2/show/NCT00751985 (Archived by WebCite at http://www.webcitation.org/68WCRLyaP)

## Introduction

Heavy alcohol intake increases the risk of numerous chronic diseases, injuries, disabilities, and death [1]. Many drinkers who do not meet diagnostic criteria for alcohol dependence or harmful drinking nonetheless consume alcohol at a level or in a pattern that increases the risk of negative health and social consequences [2]. In Denmark, it has been estimated that 20% of the adult population are heavy drinkers [3]; hence, the need to detect and intervene in the early stages of heavy drinking is obvious. Face-to-face brief interventions, which are intended as an early intervention for non-treatment-seeking, non-alcohol-dependent drinkers, have proven to be effective and have been advocated as a strategy to curb heavy drinking [4]. However, problems with feasibility and barriers to implementation have been encountered, such as a limited number of professionals who administer them and the difficulty of contacting heavy drinkers [5,6]. As a consequence, there is a gap between need and access to interventions to reduce alcohol intake. It has been estimated that as many as 80% of problem drinkers do not receive help due to a combination of missed screening opportunities and the stigma associated with alcohol treatment [7,8]. Delivering brief interventions over the Internet may overcome some of these barriers, and Internet-based interventions can reach individuals who are otherwise unwilling or not motivated to seek help [9,10]. The increasing access to the Internet in the population, currently 58% for Europe, 78% for North America, and 30% worldwide [11], and the well-documented demand for Internet-based interventions in the general public [12] mean that, if delivered broadly, Internet-based interventions could have potentially major public health implications, the main argument being that Internet-based interventions combine the scalability of a public health intervention with the capacity to deliver a personalized approach [13].

Recent systematic reviews concluded that Internet-based interventions were more effective than minimally active comparator groups at reducing alcohol intake, with a mean difference of 2–3 drinks per week [14], and found small to medium effect sizes [15-17]. However, several methodological flaws in the reviewed trials caused the authors to state that the ability to generalize about the efficacy and utility of Internet-based interventions for alcohol use is impeded, and hence it is not possible to interpret the evidence with any degree of certainty [14,17]. Unresolved questions remain, such as the need to establish which components of Internet-based interventions are effective [18]. The provision of a personalized feedback intervention that compares one’s own drinking with peers’ actual drinking has been found to increase motivation to change drinking by making individuals aware of discrepancies between their personal alcohol consumption and social norms. This approach originates in self-regulation theory and builds on the assumption that change is triggered by creating an awareness of a perceived discrepancy. Therefore, if heavy drinkers find no such discrepancy, they would view their personal behavior as being normal rather than abnormal. This personal tailored approach, also termed normative feedback, which tries to create behavioral change by targeting normative misperceptions, is assumed to be more effective at reducing drinking than is delivering standardized feedback in the form of self-help material [16,19-22].

Some drawbacks of personalized feedback intervention studies are small sample sizes, short-term follow-up, the existence of few studies in nonstudent adult populations, few comparisons with appropriate control groups, and high rates of attrition [14,16,23]. In this study, we addressed these shortcomings by comparing a single-session, Internet-based, brief personalized feedback intervention with an Internet-based, personalized, brief advice intervention against a pure control group in the context of the Danish Health Examination Survey [24]. We sought to determine whether these single-session interventions would result in a decrease in alcohol use in a non-treatment-seeking population of adult heavy drinkers. As a secondary aim, we also sought to determine whether the Internet-based brief personalized feedback intervention would have any gender-specific effects, especially since differential effectiveness between genders in non-Internet brief interventions remains ambiguous [4,25,26] and because few Internet-based intervention studies present data separately for men and women.

## Methods

**Setting**

The Danish Health Examination Survey was carried out by the National Institute of Public Health, University of Southern Denmark, in 13 municipalities in 2007/2008. The Danish Health Examination Survey focused primarily on diet, smoking, alcohol, and physical activity and consisted of an Internet-based questionnaire and a health examination. In this study, we used data from the Internet-based questionnaire from 12 of the 13 municipalities. All adult inhabitants in 12 municipalities were invited to complete the Internet-based questionnaire (n = 401,607). The sample was drawn from the adult Danish population (18 years or older) using the Danish Civil Registration System, which contains information on gender, age, address, citizenship, and marital status for each individual (each Danish resident has a unique personal identification number) [27]. The questionnaire was fully or partially completed by 54,157 participants, corresponding to 13.49% of all adults in the 12 municipalities [24].

**Recruitment**

Recruitment for the study began in September 2008. Follow-up started in February 2009 and ended in February 2010.

**Study Design**

The study was a 3-arm randomized controlled trial.

**Participants**

Invitees to the Danish Health Examination Survey received a letter inviting them to participate by completing an Internet-based questionnaire containing questions on their sociodemographic characteristics, self-reported health status, living conditions, and health behavior including alcohol consumption. The baseline questionnaire was completed at the respondent’s home. In 7 of the 12 municipalities, the questionnaire was supplemented with questions to test the willingness of respondents to change their health behavior in four domains: weight, diet, smoking, and alcohol (n = 33,554 completed these questions). The alcohol questions were beverage specific (beer, wine, fortified wine, or spirits) and asked for amount consumed each day during a typical week. Additionally, the Alcohol Use Disorders Identification Test questions 3–10 were included (timeframe: preceding 12 months) [28]. Respondents who had provided an email address (75% of the population) and whose weekly alcohol consumption was above the recommended maximum drinking limit, as stated by the Danish National Board of Health (14 drinks = 168 g of alcohol for women, 21 drinks = 252 g for men), were eligible for the study. One standard drink corresponds to 12 g of pure alcohol [29]. Heavy drinking was defined as 168 g or more of alcohol/week for women and 252 g/week or more for men. Binge drinking was defined as drinking 5 or more drinks on a single occasion both for men and women.

**Interventions**

**Figure 1:**
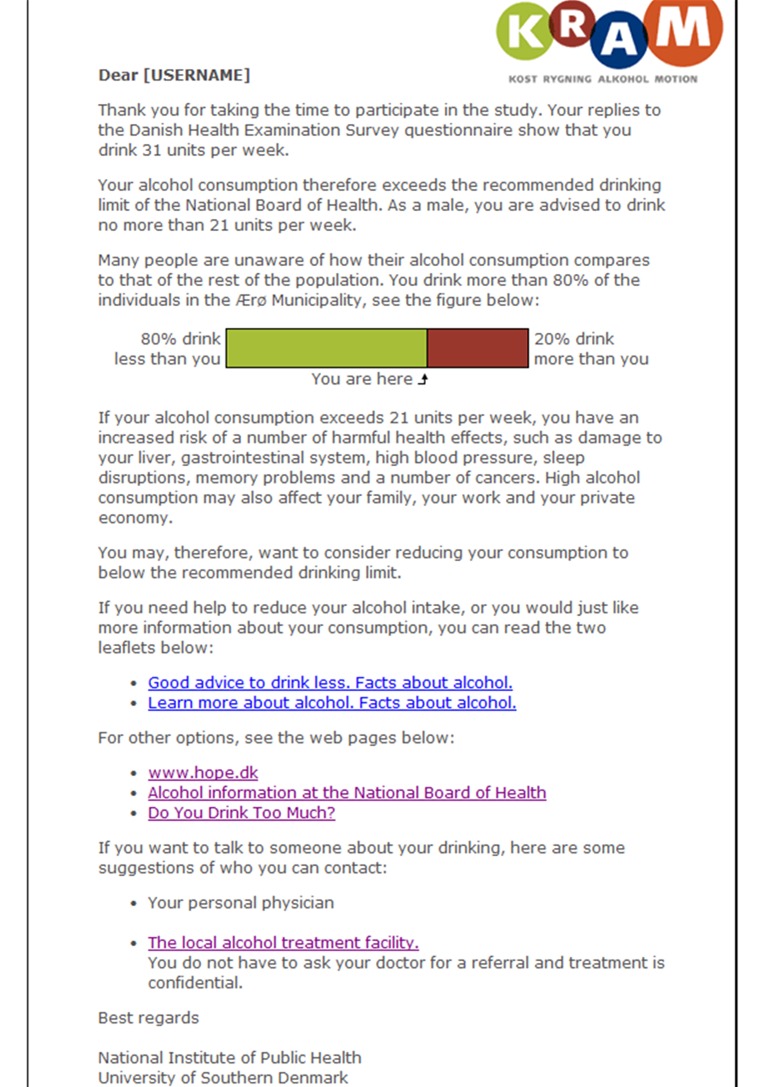
Screenshot of the Internet-based brief personalized feedback intervention.

The Internet-based brief personalized feedback intervention was a fully automated, single-session intervention; it was displayed in a single screenshot and addressed to the participant by name. It consisted of a summary of the participant’s weekly consumption, a comparison of the weekly consumption with the maximum drinking limit, and a graphical comparison of the participant’s consumption with the average level in the municipality (gender specific). The Internet-based brief personalized feedback intervention also included information about the risks to health and social relationships linked to heavy drinking, as well as links for further self-help material and a local alcohol treatment facility (see Figure 1).

**Figure 2:**
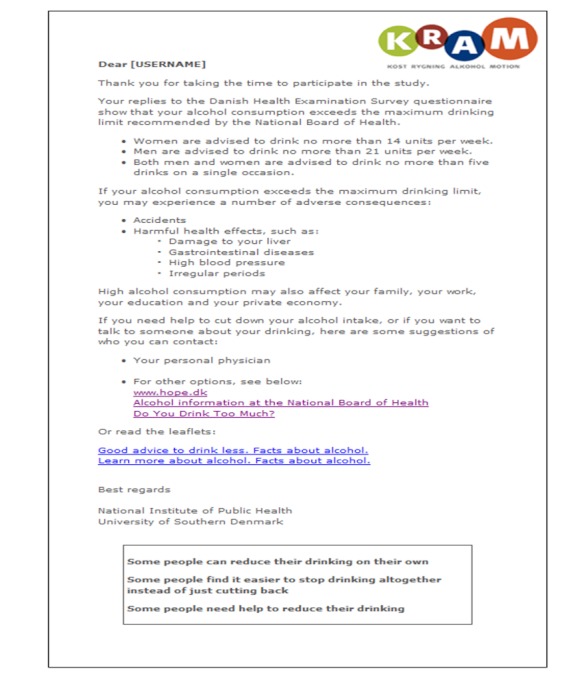
Screenshot of the Internet-based personalized brief advice intervention.

The Internet-based personalized brief advice intervention was a fully automated single-session intervention and was displayed in a single screenshot and addressed to the participant by name. It informed the participant that his or her alcohol consumption exceeded the recommended maximum drinking limit, followed by information about the health and social risks associated with heavy drinking, as well as links for further standardized self-help material and a local alcohol treatment facility (see Figure 2).What distinguishes the Internet-based brief personalized feedback intervention from the Internet-based personalized brief advice intervention is the normative component and the summary of the participant’s weekly alcohol consumption. Common to the two interventions is the information on the adverse effects of heavy drinking, advice to cut down, and links for further material (see Multimedia Appendix 1).

**Figure 3:**
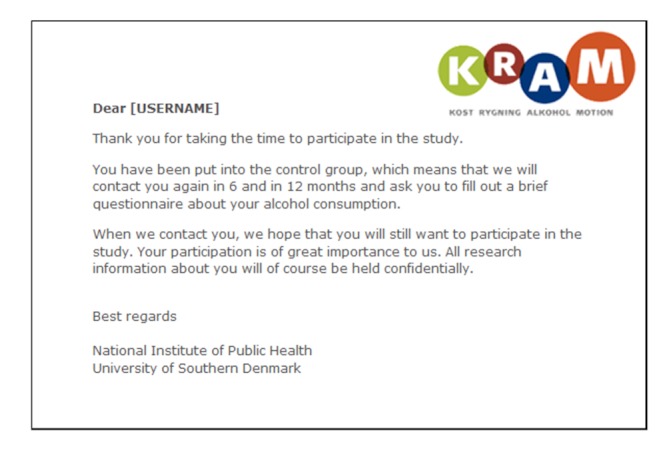
Screenshot of the control group condition.

Participants in the control group received a single screenshot that explained that being randomly selected for the control group implied no intervention and follow-up after 6 and 12 months (see Figure 3).

Individuals who consumed less than the maximum drinking limit did not receive any feedback or interventions.

**Procedure**

After completing the Internet-based Danish Health Examination Survey questionnaire, invitees were automatically screened and heavy drinkers were identified. Heavy drinkers received an email inviting them to participate in the intervention study. By clicking on a link in the email, invitees were directed to a secure website where, after entering a username and personal access code (provided in the Danish Health Examination Survey invitation letter), they were directed to another website that explained the study (see Multimedia Appendix 1). After providing their online consent, participants were automatically randomly assigned and directed to a new personalized website that presented one of the interventions or control, which was displayed immediately on the screen.

**Randomization**

Eligible persons were randomly assigned and enrolled into the Internet-based brief personalized feedback intervention, the Internet-based personalized brief advice intervention, or the control group by the Web server software, which was implemented by a technician who was not involved in the recruitment process. Blinding was not feasible. Participants did not know which of the two interventions was the intervention of interest. Prior to randomization, all three groups were informed about the purpose of the study and the nature of the control group (see Multimedia Appendix 1).

**Outcome Measure and Follow-up**

There was one planned primary analysis: overall reduction in alcohol use; and one post hoc secondary analysis: gender-specific reductions in alcohol use. The outcome measure was specified a priori and was based on self-reported drinking each day during a typical week and included beverage-specific questions (beer, wine, fortified wine, and spirits). The follow-up at 6 and 12 months contained the same alcohol items included in the baseline questionnaire and was conducted using an Internet-based questionnaire that participants accessed using a link provided in an email. The follow-up at 12 month was also supplemented by a letter containing the questionnaire, which the participants could answer if they did not respond to the email.

**Power Estimates**

The sample size was calculated based on a meta-analysis of Internet-based interventions, in which a mean difference of 2–3 drinks (26 g of alcohol) per week was found [14], and based on non-Internet-based interventions meta-analyses, where a 12% to 15% reduction in the previous week’s alcohol consumption was found, relative to a baseline consumption of approximately 300 g/week [4,30]. We anticipated a decrease in alcohol consumption of approximately 15% for the Internet-based brief personalized feedback intervention, 10% for the Internet-based personalized brief advice intervention, and 5% for the control group. Decreases in control groups in Internet-based interventions and non-Internet-based interventions have been substantial. However, this decrease has not been quantified in meta-analyses due to the highly variable content of control groups in both Internet-based interventions and face-to-face brief interventions [31]. Assuming that the standard deviation was equal to a third of the expected baseline consumption, we estimated that 182 participants in each group would be needed to give the trial 80% power to detect an effect of the Internet-based brief personalized feedback intervention versus the control group of this size at the 5% level of significance. To detect an effect of the Internet-based personalized brief advice intervention versus the control group, we estimated that 726 participants in each group would be needed. A target sample size of more than 1200 enrollees was deemed necessary to allow for substantial attrition.

**Statistical Analysis**

The primary and secondary analyses were based on the intention-to-treat principle and concerned the mean difference in changes in alcohol consumption between the two intervention groups and the control group.

We carried out analyses using Stata version 11.2 (StataCorp LP, College Station, TX, USA). Quantitative variables were described by the mean and standard deviation, by the median and its interquartile range, or by its 95% confidence interval (CI). In all tests, we chose *P *< .05 as the level of significance.

The residuals were approximately normally distributed. Hence, to examine changes over time and to account for the multiple time measurements, we analyzed data by using a multilevel mixed model, using the *xtmixed *procedure. The model examined fixed effects for alcohol consumption, group, gender, and month and a random intercept to account for clustering within each participant. The model also included an interaction term between intervention group and month, allowing for differences in the intervention effect between follow-up assessments [32]. The fixed effect of most interest was the month × group interaction effect, which indicated the difference between intervention groups and the control group as a change in alcohol consumption over time.

The Kruskal-Wallis nonparametric test for continuous variables was used to compare the three groups in the secondary analyses.

For the loss to follow-up analysis, we used the chi-square test and the Kruskal-Wallis nonparametric test for continuous variables to compare the baseline characteristics between those followed up and those lost to follow-up. In a preplanned analysis, we used multiple imputation to take attrition into account for participants who did not complete the 6- or 12-month follow-up. Multiple imputation allows for the uncertainty about the missing data by creating several different plausible imputed data sets and appropriately combining results obtained from each (we generated 20 data sets), which often provides a more reliable approach than complete case analysis in the presence of missing data [23,33]. For this we used the *mi impute mvn *procedure, which uses multivariate normal regression for continuous data and assumes that data are missing at random [34]. As sensitivity analyses, we also report results from (1) an analysis of all available results without the imputation of missing data (completers-only analysis), and (2) an analysis with simple imputation (last observation carried forward) assuming that nonresponders had no change in their alcohol consumption.

## Results

**Participant Flow**

**Figure 4:**
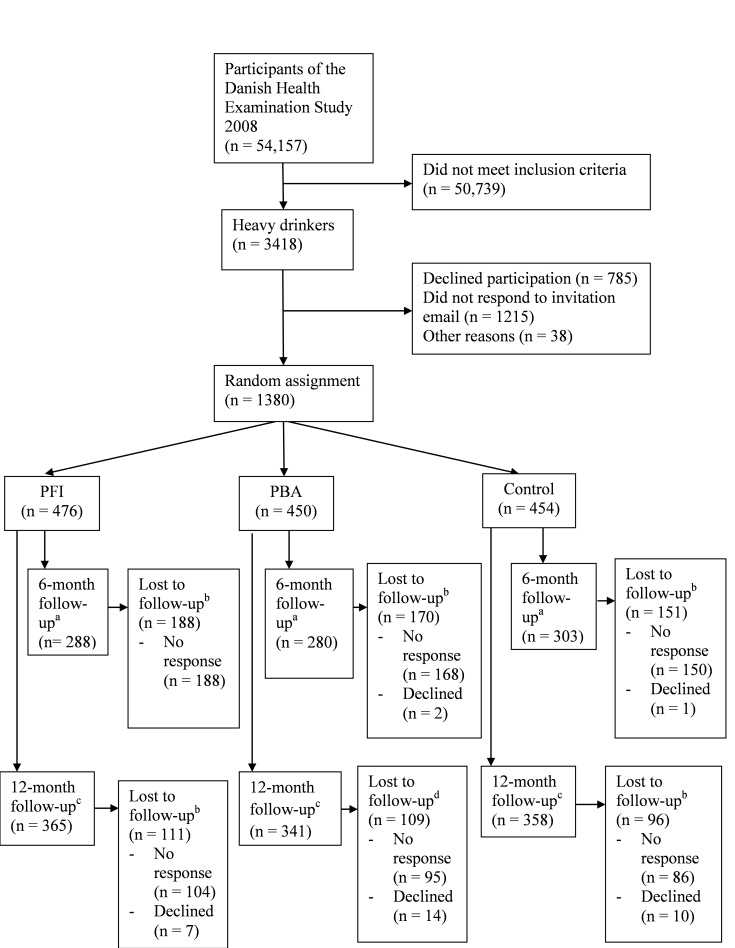
Flow of participants through the study. PBA = Internet-based personalized brief advice intervention, PFI = Internet-based brief personalized feedback intervention. ^a^Follow-up took place by means of two emails. ^b^No response and declined are subsets of lost to follow-up. ^c^ Follow-up took place by means of two emails and two letters.

Of the 54,157 screened individuals, 3418 (6.31%) were heavy drinkers. Of these, 785 (23.0%) declined participation, and 1215 (35.6%) did not respond to the invitation email. In total, 1380 (40.37%) individuals accepted participation and were randomly assigned into the Internet-based brief personalized feedback intervention (n = 476), the Internet-based personalized brief advice intervention (n = 450), or the control group (n = 454). The 6-month follow-up was completed by 871 (63.1% of enrolled participants) individuals and the 12-month follow-up was completed by 1064 (77.10%) individuals (Figure 4).

**Baseline Data**

At baseline, men consumed a mean of 32 drinks/week and women 21 drinks/week. During the previous year, 49.9% (n = 380) of the men had been binge drinking once a week or more often, while among women the corresponding figure was 25.5% (n = 158) (Table 1). At baseline, 384 (46.2%) individuals answered “yes” or “yes, maybe” to the question “Do you want to cut down on your drinking?”, 319 (38.4%) answered “no”, and 128 (15.4%) did not respond (831 individuals received this question). The median age was 58 years, 55.1% (n = 761) were men, 51.7% (n = 714) had more than 15 years of education, 53.1% (n = 733) were employed, and 69.6% (n = 961) were married or cohabiting. Among the participants, 10% (n = 139) were daily smokers and 12% (n = 161) were heavy smokers (more than 15 cigarettes a day). There were no significant differences between randomized groups for any baseline characteristic.

**Table 1:** Baseline characteristics of participants randomly assigned to Internet-based brief personalized feedback intervention (PFI), Internet-based personalized brief advice intervention (PBA), or control group in The Danish Health Examination Survey 2008.

Characteristic			PFI	PBA	Control
**Men**					
	No.		271	246	244
	Age (years), median (IQR^a^)		61 (50–66)	59 (49–65)	60 (51–65)
	Alcohol intake (drinks/week)^b^, mean (SD)		32.8 (16.9)	32.7 (14.0)	31.3 (10.3)
	Binge drinking, n (%)^c^		137 (50.5%)	118 (48.0%)	125 (51.2%)
	Education level (years), n (%)				
		<10	11 (4%)	19 (8%)	10 (4%)
		10–12	65 (24%)	55 (22%)	59 (24%)
		13–14	45 (17%)	50 (20%)	55 (23%)
		15+	149 (54.9%)	117 (47.5%)	118 (48.3%)
	Employed, n (%)		146 (53.8%)	121 (49.1%)	144 (59.0%)
	Smoking, n (%)				
		Daily	31 (11%)	29 (12%)	23 (9%)
		Heavy^d^	40 (15%)	29 (12%)	26 (11%)
	Married or cohabiting, n (%)		198 (73.1%)	172 (69.9%)	185 (75.8%)
	Motivated to reduce alcohol use, n (%)^e^				
		Yes	19 (7%)	26 (11%)	22 (9%)
		Yes, maybe	53 (20%)	41 (17%)	47 (19%)
		No	56 (21%)	56 (23%)	57 (23%)
**Women**					
	No.		205	204	210
	Age (years), median (IQR)		54 (41–62)	56 (46–63)	56 (44–62)
	Alcohol intake (drinks/week), mean (SD)		20.9 (7.0)	21.5 (9.0)	21.3 (8.2)
	Binge drinking, n (%)		50 (24%)	55 (27%)	53 (25%)
	Education level (years), n (%)				
		<10	10 (5%)	10 (5%)	12 (6%)
		10–12	55 (27%)	41 (20%)	45 (21%)
		13–14	32 (16%)	39 (19%)	41 (20%)
		15+	107 (52.2%)	112 (54.9%)	111 (52.9%)
	Employed, n (%)		107 (52.2%)	116 (56.9%)	99 (47%)
	Smoking, n (%)				
		Daily	20 (8%)	19 (9%)	17 (8%)
		Heavy	26 (13%)	18 (9%)	22 (10%)
	Married or cohabiting, n (%)		129 (62.9%)	135 (66.2%)	142 (67.6%)
	Motivated to reduce alcohol use, n (%)				
		Yes	24 (12%)	21 (10%)	23 (11%)
		Yes, maybe	33 (16%)	32 (16%)	43 (20%)
		No	49 (24%)	48 (24%)	53 (25%)

^a ^Interquartile range.

^b ^Number of standard drinks in a typical week.

^c ^Drinking 5 or more drinks per occasion at least once a week.

^d ^Smoking more than 15 cigarettes a day.

^e ^Numbers do not sum to 100% due to missing data.

**Loss to Follow-up Analysis**

We compared participants lost to follow-up (n = 509, 37% at 6 months and n = 316, 23% at 12 months) by intervention group with those who participated in the follow-up in terms of baseline characteristics.

Participants lost to follow-up were significantly more likely to be heavy smokers, less likely to have a high level of education (15+ years), and more likely to have a low level of education (10–12 years). Furthermore, participants lost to follow-up were more likely to be unmotivated to cut down their drinking (Table 2 and Table 3).

**Table 2:** Comparison of characteristics of participants randomly assigned to Internet-based brief personalized feedback intervention (PFI), Internet-based personalized brief advice intervention (PBA), or control group at baseline between those followed up after 6 months and those lost at 6-month follow-up.^a^

Characteristic		Followed up after 6 months			Lost to follow-up at 6 months					
		PFI (n = 288)	PBA (n = 280)	Control (n = 303)	PFI (n = 188)	*P* value^b^	PBA (n = 170)	*P* value^c^	Control (n = 151)	*P* value^d^
Men, n (%)		161 (55.9%)	155 (55.4%)	165 (54.5%)	110 (58.5%)	.57	91 (54%)	.71	79 (52%)	.67
Women, n (%)		127 (44.1%)	125 (44.6%)	138 (45.5%)	78 (42%)		79 (47%)		72 (48%)	
Age (years), median (IQR)^e^		58 (46–65)	58 (48–64)	60 (48–64)	58 (47–65)	.46	57 (47–65)	.89	56 (46–63)	.04
Alcohol intake, mean (SD)^f^		28.0 (16.7)	27.9 (12.9)	26.1 (9.6)	27.1 (11.1)	.85	27.0 (13.7)	.43	27.9 (12.9)	.35
Binge drinking, n (%)^g^		114 (39.6%)	117 (41.8%)	119 (39.3%)	73 (39%)	.85	56 (33%)	.05	59 (39%)	.98
**Education level (years), n (%)** ^h^						.03		.28		.65
	<10	13 (5%)	17 (6%)	15 (5%)	8 (4%)		12 (7%)		7 (5%)	
	10–12	66 (23%)	52 (19%)	64 (21%)	54 (29%)		44 (26%)		40 (26%)	
	13–14	38 (13%)	56 (20%)	66 (22%)	39 (21%)		33 (19%)		30 (20%)	
	15+	170 (59.0%)	150 (53.6%)	156 (51.5%)	86 (46%)		79 (47%)		73 (48%)	
Employed, n (%)		155 (53.8%)	152 (54.3%)	157 (51.8%)	98 (52%)	.80	85 (50%)	.37	86 (57%)	.30
**Smoking, n (%)**						.02		.01		.12
	Daily	31 (11%)	38 (14%)	28 (9%)	20 (11%)		10 (6%)		12 (8%)	
	Heavy^i^	29 (10%)	21 (8%)	25 (8%)	37 (20%)		26 (15%)		23 (15%)	
Married or cohabiting, n (%)		203 (70.5%)	190 (67.9%)	219 (72.3%)	124 (66.0%)	.04	117 (68.8%)	.12	108 (71.5%)	.28
**Motivated to reduce alcohol use, n (%)** ^h^						.02		.56		.47
	“Yes” or “yes, maybe”	87 (56%)	84 (47%)	99 (48%)	42 (38%)		36 (38%)		36 (41%)	
	“No”	55 (36%)	69 (39%)	76 (37%)	50 (45%)		35 (37%)		34 (39%)	

^a^ *P *values for categorical variables by chi-square test and for continuous variables by Kruskal-Wallis test.

^b ^Participants in the PFI group who participated in follow-up versus those lost to follow-up.

^c ^Participants in the PBA group who participated in follow-up versus those lost to follow-up.

^d ^Participants in the control group who participated in follow-up versus those lost to follow-up.

^e ^Interquartile range.

^f ^Number of standard drinks in a typical week.

^g ^Drinking 5 or more drinks per occasion at least once a week.

^h ^Numbers do not sum to 100% due to missing data.

^i ^Smoking more than 15 cigarettes a day.

**Table 3:** Comparison of characteristics of participants randomly assigned to Internet-based brief personalized feedback intervention (PFI), Internet-based personalized brief advice intervention (PBA), or control group at baseline between those followed up after 12 months and those lost at 12-month follow-up.^a^

Characteristic		Followed up after 12 months			Lost to follow-up 12 months					
		PFI (n = 365)	PBA (n = 341)	Control (n = 358)	PFI (n = 111)	*P* value^b^	PBA (n = 109)	*P* value^c^	Control (n = 96)	*P* value^d^
Men, n (%)		209 (57.3%)	193 (56.6%)	196 (54.8%)	62 (56%)	.79	53 (49%)	.15	48 (50%)	.41
Women, n (%)		156 (42.7%)	148 (43.4%)	162 (45.3%)	49 (44%)		56 (51%)		48 (50%)	
Age (years), median (IQR)^e^		58 (47–65)	58 (48–65)	60 (49–65)	57 (47–64)	.69	55 (46–63)	.11	54 (44–61)	<.01
Alcohol intake, mean (SD)^f^		27.7 (15.6)	27.5 (13.5)	26.4 (9.9)	27.6 (11.5)	.72	28.0 (12.3)	.47	27.9 (12.8)	.80
Binge drinking, n (%)^g^		139 (38.1%)	133 (39.0%)	131 (36.6%)	48 (43%)	.34	40 (37%)	.70	47 (49%)	.03
**Education level (years), n (%)** ^h^						<.01		.10		.09
	<10	13 (4%)	22 (6)	19 (5)	8 (7)		7 (6)		3 (3)	
	10–12	87 (24%)	66 (19)	77 (22)	33 (30)		30 (28)		27 (28)	
	13–14	50 (14%)	63 (18)	70 (20)	27 (24)		26 (24)		26 (27)	
	15+	213 (58.4%)	184 (54.0%)	190 (53.1%)	43 (39%)		45 (41%)		39 (41%)	
Employed, n (%)		196 (53.7%)	178 (52.2%)	184 (51.4%)	57 (51%)	.65	59 (54%)	.70	59 (61%)	.07
**Smoking, n (%)**						.02		.23		.44
	Daily	38 (10%)	39 (11%)	35 (10%)	13 (12%)		9 (8%)		5 (5%)	
	Heavy^i^	41 (11%)	30 (9%)	36 (10%)	25 (23%)		17 (16%)		12 (13%)	
Married or cohabiting, n (%)		258 (70.7%)	231 (67.7%)	258 (72.1%)	69 (62%)	.19	76 (70%)	.81	69 (72%)	.70
**Motivated to reduce alcohol use, n (%)** ^h^						.02		.07		.26
	“Yes” or “yes, maybe”	108 (53%)	91 (43%)	112 (48%)	21 (33%)		29 (47%)		23 (40%)	
	“No”	74 (36%)	89 (42%)	85 (36%)	31 (49%)		15 (24%)		25 (44%)	

^a^ *P *values for categorical variables by chi-square test and for continuous variables by Kruskal-Wallis test.

^b ^Participants in the PFI group who participated in follow-up versus those lost to follow-up.

^c ^Participants in the PBA group who participated in follow-up versus those lost to follow-up.

^d ^Participants in the control group who participated in follow-up versus those lost to follow-up.

^e ^Interquartile range.

^f ^Number of standard drinks in a typical week.

^g ^Drinking 5 or more drinks per occasion at least once a week.

^h ^Numbers do not sum to 100% due to missing data.

^i ^Smoking more than 15 cigarettes a day.

### Outcomes

Table 4 and Table 5 present the intervention effects of the Internet-based brief personalized feedback intervention and the Internet-based personalized brief advice intervention, with and without imputation for missing values. The intervention effects indicate the additional difference in change in alcohol consumption in the intervention groups compared with the control group.

In the primary analysis, using multiple imputation, the intervention effects of the Internet-based brief personalized feedback intervention were –1.8 drinks/week after 6 months and –1.4 drinks/week after 12 months and were nonsignificant (95% CI –4.0 to 0.3 at 6 months, –3.4 to 0.6 at 12 months). The intervention effects of the Internet-based personalized brief advice intervention were –0.5 drinks/week after 6 months and –1.2 drinks/week after 12 months and were nonsignificant (95% CI –2.7 to 1.6 at 6 months, –3.3 to 0.9 at 12 months).

A sensitivity analysis without multiple imputation for missing values (completers-only analysis) showed that the intervention effects of the Internet-based brief personalized feedback intervention were –3.9 drinks/week after 6 months and –2.3 drinks/week after 12 months; these effects were significant (95% CI –5.8 to –2.0 at 6 months, –4.1 to –0.5 at 12 months). The Internet-based personalized brief advice intervention had no significant intervention effects.

A sensitivity analysis with simple imputation (last observation carried forward) yielded similar results to the completers-only analysis, but the differences were less pronounced.

For the control group, the overall difference between the baseline and 6-month follow-up was –4.6 drinks/week, and this difference was significant (95% CI –6.1 to –3.1). Corresponding figures for 12-month follow-up were –5.5 (95% CI –7.0 to –4.1). The two sensitivity analyses produced similar results (Table 4 and Table 5).

**Table 4:** Intervention effects on drinks/week based on random intercept model with and without imputation for missing values.

		With multiple imputation for missing values^a^			Without multiple imputation for missing values^b^		
		Drinks/week^c^	95% CI^d^	*P *value	Drinks/week	95% CI	*P *value
**Intervention effects of the PFI** ^e ^ **(month × group interaction)**							
	6 months	–1.8	–4.0 to 0.3	.09	–3.9	–5.8 to –2.0	<.001
	12 months	–1.4	–3.4 to 0.6	.16	–2.3	–4.1 to –0.5	.01
**Intervention effects of the PBA** ^f ^ **(month × group interaction)**							
	6 months	–0.5	–2.7 to 1.6	.62	–1.4	–3.3 to 0.6	.17
	12 months	–1.2	–3.3 to 0.9	.28	–1.5	–3.3 to 0.3	.10
**Difference between baseline and follow-up for control group**							
	6 months	–4.6	–6.1 to –3.1	<.001	–4.8	–6.1 to –3.4	<.001
	12 months	–5.5	–7.0 to –4.1	<.001	–5.8	–7.1 to –4.6	<.001

^a ^Based on 20 imputed datasets.

^b ^Based on 871 individuals after 6 months and 1064 after 12 months.

^c ^Mean number of standard drinks in a typical week.

^d ^Confidence interval.

^e ^Internet-based brief personalized feedback intervention.

^f ^Internet-based personalized brief advice intervention.

**Table 5:** Intervention effects on drinks/week based on random intercept model with simple imputation for missing values (last observation carried forward).^a^

		Drinks/week^b^	95% CI^c^	*P *value
**Intervention effects of the PFI** ^d ^ **(month × group interaction)**				
	6 months	–2.5	–4.0 to –1.0	<.001
	12 months	–2.0	–3.4 to –0.5	.01
**Intervention effects of the PBA** ^e ^ **(month × group interaction)**				
	6 months	–0.8	–2.3 to 0.6	.27
	12 months	–1.2	–2.7 to 0.3	.11
**Difference between baseline and follow-up for control group**				
	6 months	–2.9	–4.0 to –1.9	<.001
	12 months	–4.8	–5.9 to –3.8	<.001

^a ^Based on 1380 individuals after 6 and 12 months.

^b ^Mean number of standard drinks in a typical week.

^c ^Confidence interval.

^d ^Internet-based brief personalized feedback intervention.

^e ^Internet-based personalized brief advice intervention.

**Figure 5:**
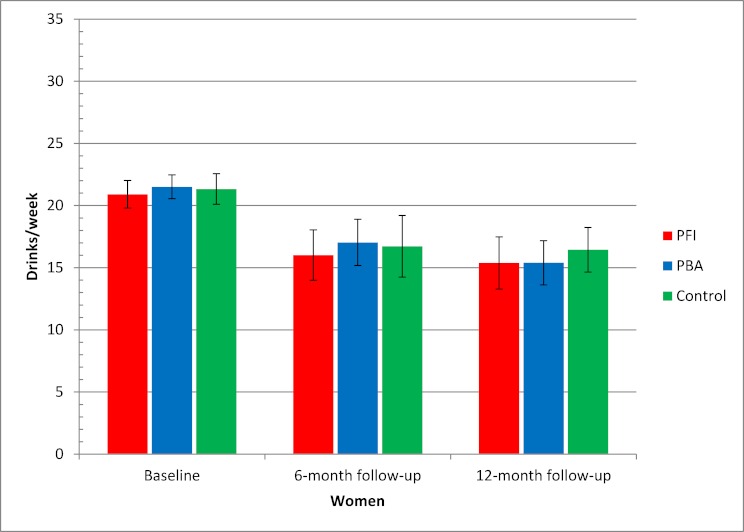
Alcohol consumption at baseline and at 6- and 12-month follow-ups for women based on multiple imputation. Error bars indicate 95% confidence interval. Drinks/week = mean number of standard drinks in a typical week, PBA = Internet-based personalized brief advice intervention, PFI = Internet-based brief personalized feedback intervention.

**Figure 6:**
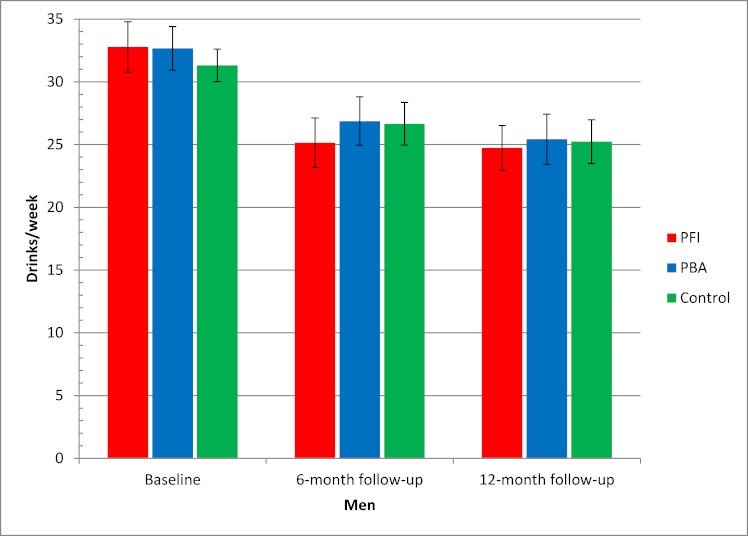
Alcohol consumption at baseline and at 6- and 12-month follow-ups for men based on multiple imputation. Error bars indicate 95% confidence interval. Drinks/week = mean number of standard drinks in a typical week, PBA = Internet-based personalized brief advice intervention, PFI = Internet-based brief personalized feedback intervention.

Figure 5 and Figure 6 show secondary post hoc analyses comprising descriptive statistics for alcohol consumption, by gender and group, at baseline and at 6- and 12-month follow-ups using multiple imputation. Consumption among women decreased from a mean baseline level of 21.0 drinks/week to 16.7 drinks/week for the control group (95% CI 14.7–18.8), 16.0 drinks/week for the Internet-based brief personalized feedback intervention (95% CI 14.2–17.9), and 17.0 for the Internet-based personalized brief advice intervention (95% CI 14.6–19.5) after 6 months (Figure 5). Consumption among men decreased from a mean baseline level of 32.0 drinks/week to 26.7 drinks/week for the control group (95% CI 25.0–28.4), 25.1 drinks/week for the Internet-based brief personalized feedback intervention (95% CI 23.2–27.1), and 26.9 drinks/week for the Internet-based personalized brief advice intervention (95% CI 24.9–28.8) after 6 months (Figure 6). Figures for the 12-month follow-up were approximately similar (Figure 5 and Figure 6).

**Figure 7:**
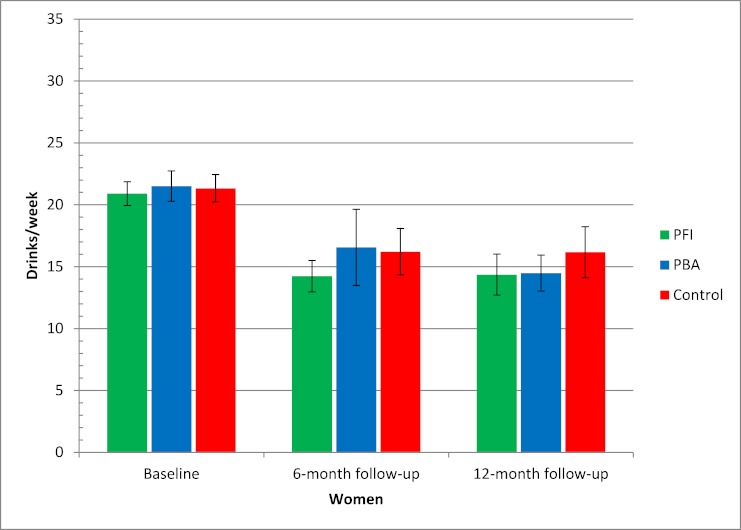
Alcohol consumption at baseline and at 6- and 12-month follow-ups for women based on completers-only analysis. n = 390 after 6 months and 466 after 12 months. Error bars indicate 95% confidence interval. Drinks/week = mean number of standard drinks in a typical week, PBA = Internet-based personalized brief advice intervention, PFI = Internet-based brief personalized feedback intervention.

**Figure 8:**
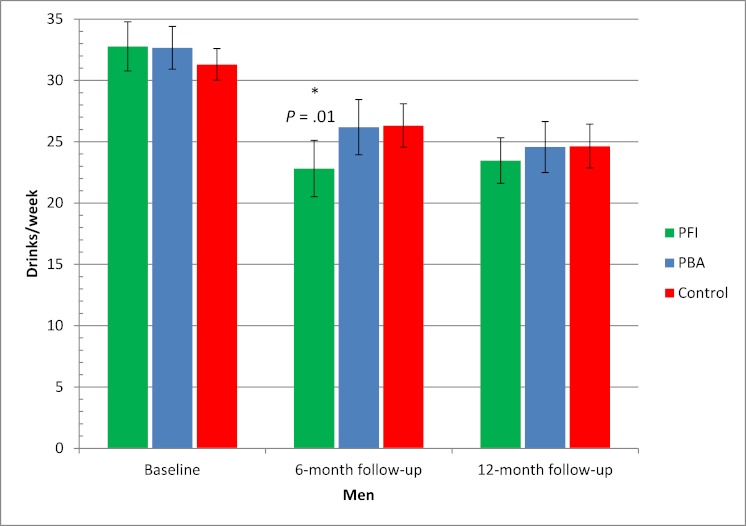
Alcohol consumption at baseline and at 6- and 12-month follow-ups for men based on completers-only analysis. n = 481 after 6 months and 598 after 12 months. Error bars indicate 95% confidence interval. Drinks/week = mean number of standard drinks in a typical week, PBA = Internet-based personalized brief advice intervention, PFI = Internet-based brief personalized feedback intervention. *P value for difference between PFI and control group (Kruskal-Wallis test).

When analyzing only those who participated in follow-up (completers-only analysis), we observed significant differences between men and women. Figure 7 and Figure 8 show that the Internet-based brief personalized feedback intervention seemed to have a significant effect only on men, with a difference of 3.5 drinks/week between the Internet-based brief personalized feedback intervention and the control group at 6-month follow-up (*P *= .01) (Figure 8).

## Discussion

**Key Findings**

In this randomized controlled trial, the primary analysis provided no evidence that an Internet-based brief personalized feedback intervention was effective in reducing drinking in an adult population of heavy drinkers. The intervention effect of the Internet-based brief personalized feedback intervention was approximately 2 drinks/week and nonsignificant, but highly significant in the sensitivity analyses with an intervention effect of approximately 3 drinks/week for the Internet-based brief personalized feedback intervention. In a post hoc secondary completers analysis of men, we found a significant difference of 3.5 drinks/week between the Internet-based brief personalized feedback intervention and the control group at the 6-month follow-up. However, it must be stressed that when interpreting the results from the completers analysis, we are dealing with a self-selected sample and no longer an unbiased sample from a randomized trial, and hence no clear conclusions regarding the efficacy of the intervention can be drawn. From baseline to 6- and 12-month follow-ups, alcohol consumption declined significantly in both intervention groups and the control group by approximately 6 drinks/week.

**Possible Mechanism and Explanations for the Findings**

When interpreting these results, other factors must be borne in mind that could have contributed to the null findings. Participating in a health examination survey may have motivated participants to change their health behavior, which may have contributed to the decrease in alcohol consumption. Of particular interest in this context is the fact that 46% were motivated to change their alcohol consumption, while 38% were not (that is, of the subsample of 831 persons who were given questions about motivation). The nonblinded nature of the study, and hence the assessment effects (intervention effects of the research procedures), could also explain part of the significant reductions in all groups from the baseline to follow-up [35,36]. These reductions could also be related to regression to the mean, social desirability bias, and historical changes in alcohol consumption. The implications of the above-mentioned effects, if they occurred, are important because, when assessment has a therapeutic benefit or when regression to the mean occurs, the experimental contrast can blur. This is particularly important for brief interventions, where effect sizes are modest [37]. The above-mentioned effects may have biased the results towards the null and hence the intervention effect may be underestimated [38]. It should also be noted that the null finding for the Internet-based personalized brief advice intervention must be interpreted in light of the insufficient sample size to detect an effect of the Internet-based personalized brief advice intervention.

**Results in Relation to Other Studies**

The null finding in our study is not unusual and mirrors the findings in two recently published trials [39,40]. However, these trials did not include a pure control group, and their intervention websites were somewhat more extensive than the Internet-based brief personalized feedback intervention and Internet-based personalized brief advice intervention used in our study. In fact, many studies have used much more extensive interventions than the very brief Internet-based brief personalized feedback intervention we used. For example, Riper et al used a multicomponent, interactive self-help intervention with a recommended treatment period of 6 weeks [41]. Other studies, such as that of Cunningham et al, recruited participants from a general population telephone survey, which differs from our recruitment procedure by way of a health examination survey [42]. This is likely to have implications for the study population, with regard to generalizability, as a sample of problem drinkers recruited through a telephone survey could be hypothesized to display a broader spectrum of alcohol problems than would a sample from a health examination survey with an emphasis on lifestyle issues in relation to diet, smoking, alcohol, and physical activity [43]. The short duration of our interventions could explain why our study’s findings differ from those of three recent meta-analyses, which concluded that brief interventions based on normative feedback are more effective than those that do not include these features [14-16]. Riper et al found an effect size (Cohen *d*) of 0.22 (95% CI 0.16–0.29) for brief, single-session personalized feedback interventions [16], and Webb et all observed small but significant effects on behavior for interventions that provided automated tailored feedback, with an effect size (Cohen *d*) of 0.18 (95% CI 0.07–0.28) [15]. In terms of amount, a systematic review found a mean difference of 26 g of alcohol between computer-based interventions and minimally active comparator groups [14].

**Strengths and Limitations of the Study**

In this rigorously conducted trial, we succeeded in implementing an Internet-based intervention in a general population-based sample of heavy drinkers. The naturalistic setting of the trial (ie, participants accessed the intervention in their own homes) increases confidence in the generalizability of the results [18,42]. Another strength of the naturalistic trial design is that it elucidates important feasibility aspects of reaching a non-treatment-seeking population of heavy drinkers by email. Knowing that 36% of the invitees did not respond to the invitation email and that 23% declined participation is applicable knowledge when designing and disseminating similar interventions.

Our aim was to investigate how minimal an Internet-based intervention can be while still having an impact on drinking. Thus, the interventions were displayed in a single screenshot immediately after the participants had provided their online consent, and we avoided the problem of knowing whether the participants randomly assigned to the interventions actually used the interventions [42].

Due to our design with two intervention groups and a pure control group, this study partially supports our hypothesis that the active component in our interventions is feedback regarding one’s own drinking relative to normative standards, at least in the sensitivity analyses. This knowledge can be used in the design of future alcohol interventions. Knowing the mechanism of change would be an important contribution to the Internet-based interventions field, as the existing research in this area has been focused on college samples [44].

Attrition (37% at 6 months and 23% at 12 months) is an area of concern in our study, as it could introduce a selection bias, thereby causing imbalance among the previously randomized groups and threatening internal validity. This was partly confirmed by our analysis, which revealed differential attrition. Participants lost to follow-up from the Internet-based brief personalized feedback intervention group were more likely to be unmotivated to cut down on their drinking. Furthermore, participants lost to follow-up were more likely to be heavy smokers and to have a low level of education. The importance of dealing with this complicated picture of differential attrition is underlined by the sensitivity analyses, which revealed that an analysis of completers only, or an analysis using last observation carried forward, will probably overestimate the treatment effects. By using multiple imputation in our main analysis, we have provided a plausible estimate of the possible result if no attrition had occurred. The generalizability of the present findings is restricted due to the underrepresentation of individuals with the lowest level of education, unmarried individuals, and younger individuals in our population. However, generalizability is a common problem in much brief intervention research that deals with populations that are not representative of the population of heavy drinkers [37,45]. This was confirmed by our results, which showed that in the Danish Health Examination Survey population, 6% were heavy drinkers, compared with a 20% prevalence estimated for the Danish population as a whole [3]. Due to our recruitment of participants from a sample in which almost everyone had Internet access, Internet and computer literacy were high in our sample. When generalizing to the Danish population, the fact that 86% of Danes have Internet access should be borne in mind. The results must also be interpreted in consideration the possibility that the use of a health examination survey to proactively enlist heavy drinkers (who were not seeking help) may have resulted in a preponderance of heavy drinkers with low levels of alcohol-related harm.

We relied on measuring outcome using self-reports of alcohol consumption, which is a method that has demonstrated reasonable levels of accuracy [37,46]. We tried to minimize the bias of underreporting by asking beverage-specific questions, which has been shown to yield higher volumes of alcohol consumption than questions that only ask for total alcohol consumption [47].

**Conclusions**

In this Internet-based study, we compared the efficacy of personalized brief advice and personalized normative feedback against a pure control group in a non-treatment-seeking population of adult heavy drinkers. The main analysis lends no support to the efficacy of personalized normative feedback or personalized brief advice. However, on the grounds of the sensitivity analyses, we cautiously conclude that the personalization in conjunction with the normative feedback enhanced attention to the message in the Internet-based brief personalized feedback intervention and thus gave an indication of decreased alcohol consumption in the Internet-based brief personalized feedback intervention group. It seems that the potential of encouraging people to become more aware of the level and consequences of their drinking, and how their drinking behaviors compare with those of others in a similar social or demographic group, is an applicable insight, in both medical and public health settings, when it comes to reducing heavy drinking.

## Multimedia Appendix 1

Interactive website used for the study, including information about the study, online consent, interventions and control conditions, and screenshots of additional material linked to from the interventions. Website can be found at URL: http://d1m.dk/DANHES/ [No login needed, translated from Danish].

## Multimedia Appendix 2

CONSORT-Ehealth Checklist V1.6.1 [48].

## Abbreviations

CI: confidence interval
