# Supplementary material for: Internet-Based Brief Personalized Feedback Intervention in a Non-Treatment-Seeking Population of Adult Heavy Drinkers: A Randomized Controlled Trial
Source: J Med Internet Res. 2012 Jul 30;14(4):e98. doi: 10.2196/jmir.1883 (PMC3409578; doi:10.2196/jmir.1883)
Supplement: Supplementary file 1 [file jmir_v14i4e98_app1.ppt]

## Slide 1
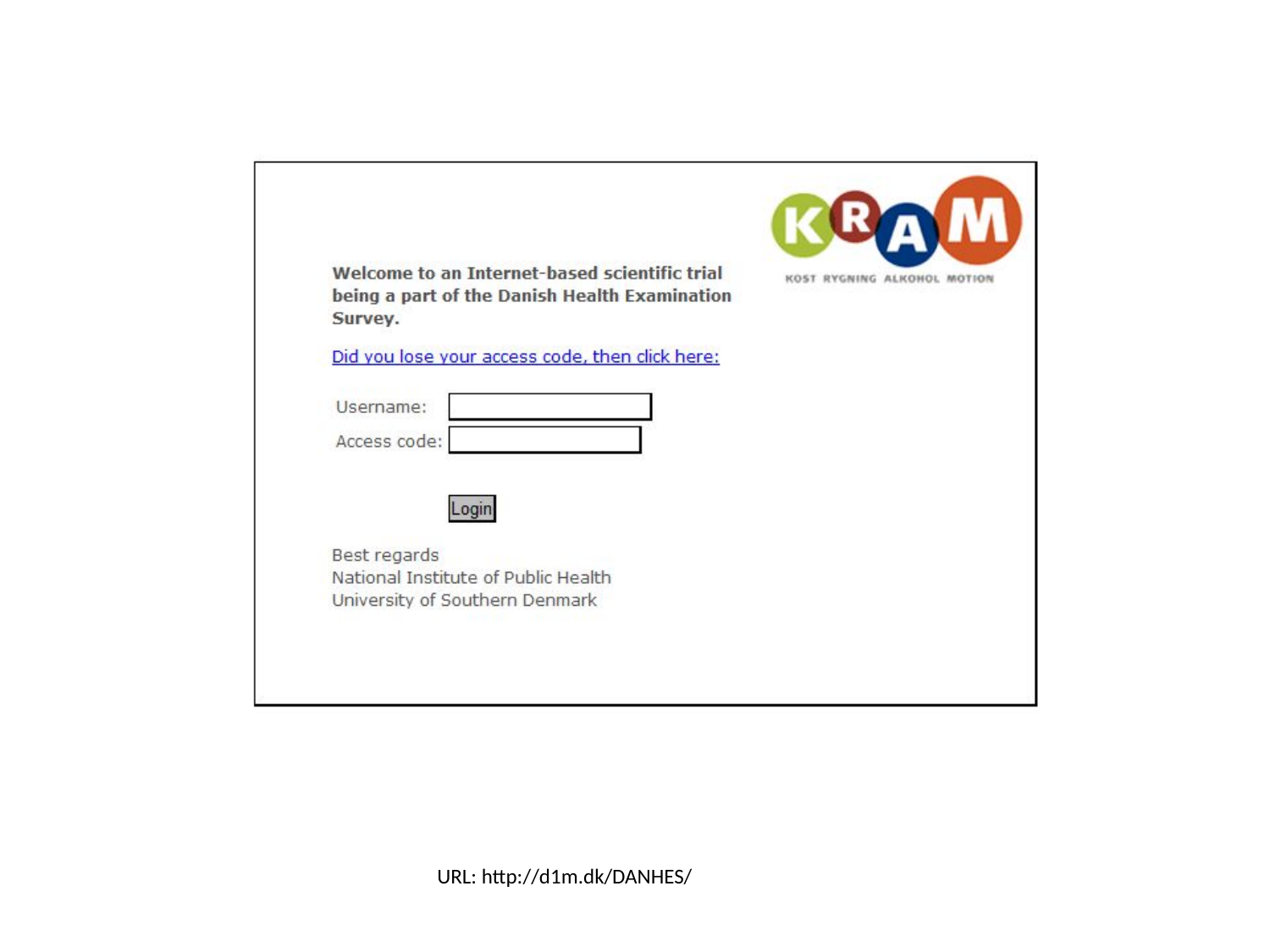

#
URL: http://d1m.dk/DANHES/

## Slide 2
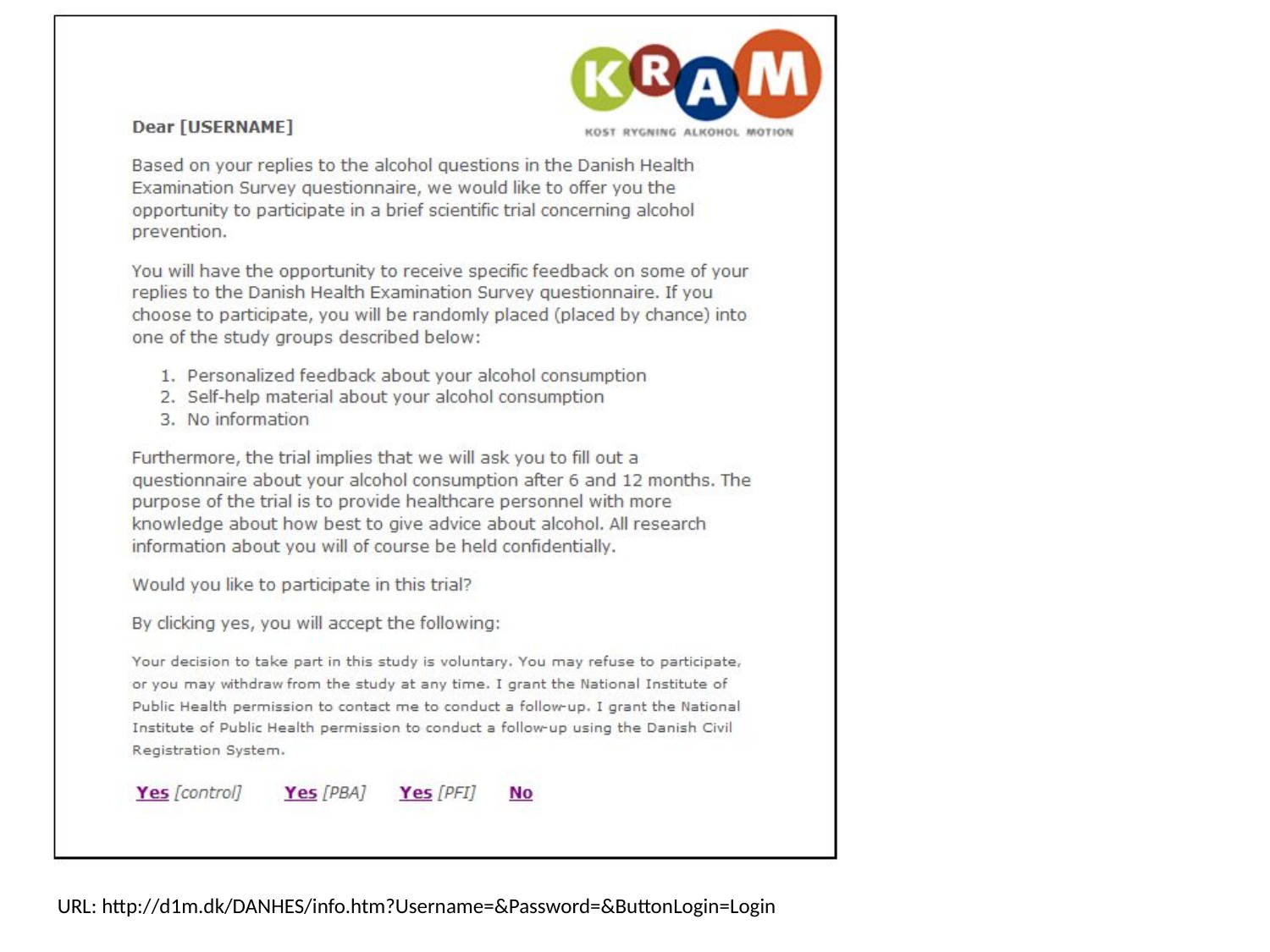

URL: http://d1m.dk/DANHES/info.htm?Username=&Password=&ButtonLogin=Login

## Slide 3
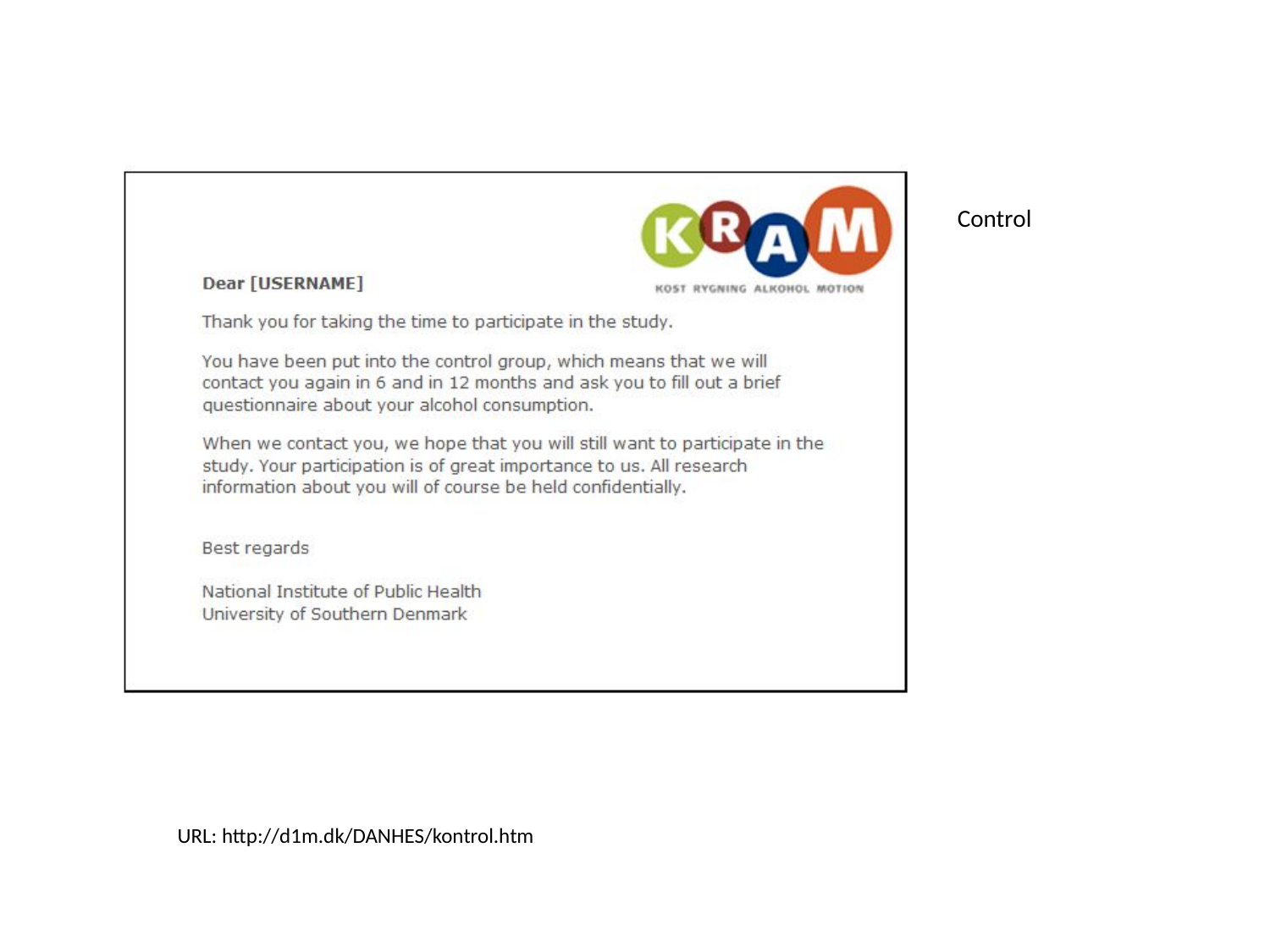

Control
URL: http://d1m.dk/DANHES/kontrol.htm

## Slide 4
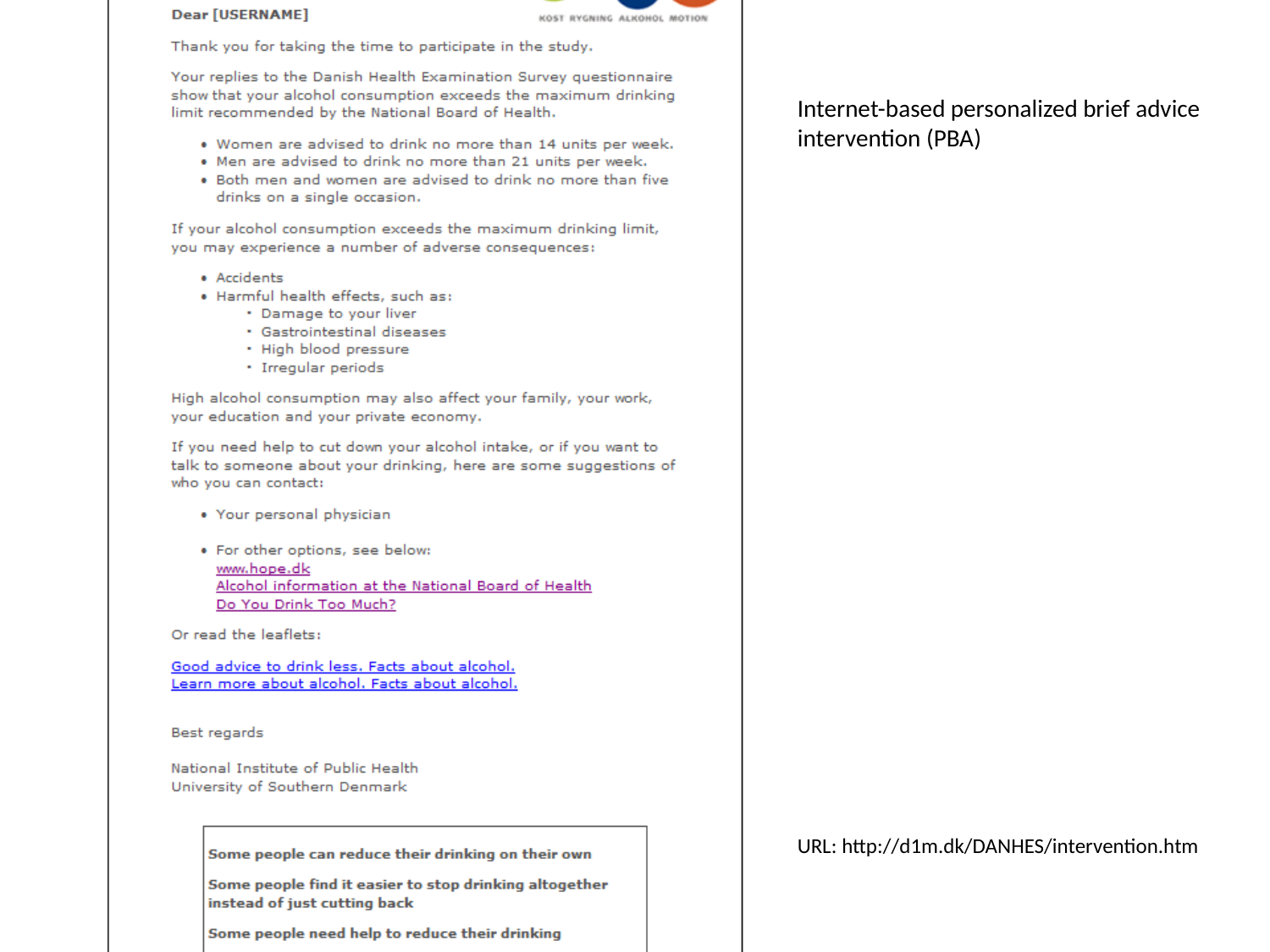

Internet-based personalized brief advice intervention (PBA)
URL: http://d1m.dk/DANHES/intervention.htm

## Slide 5
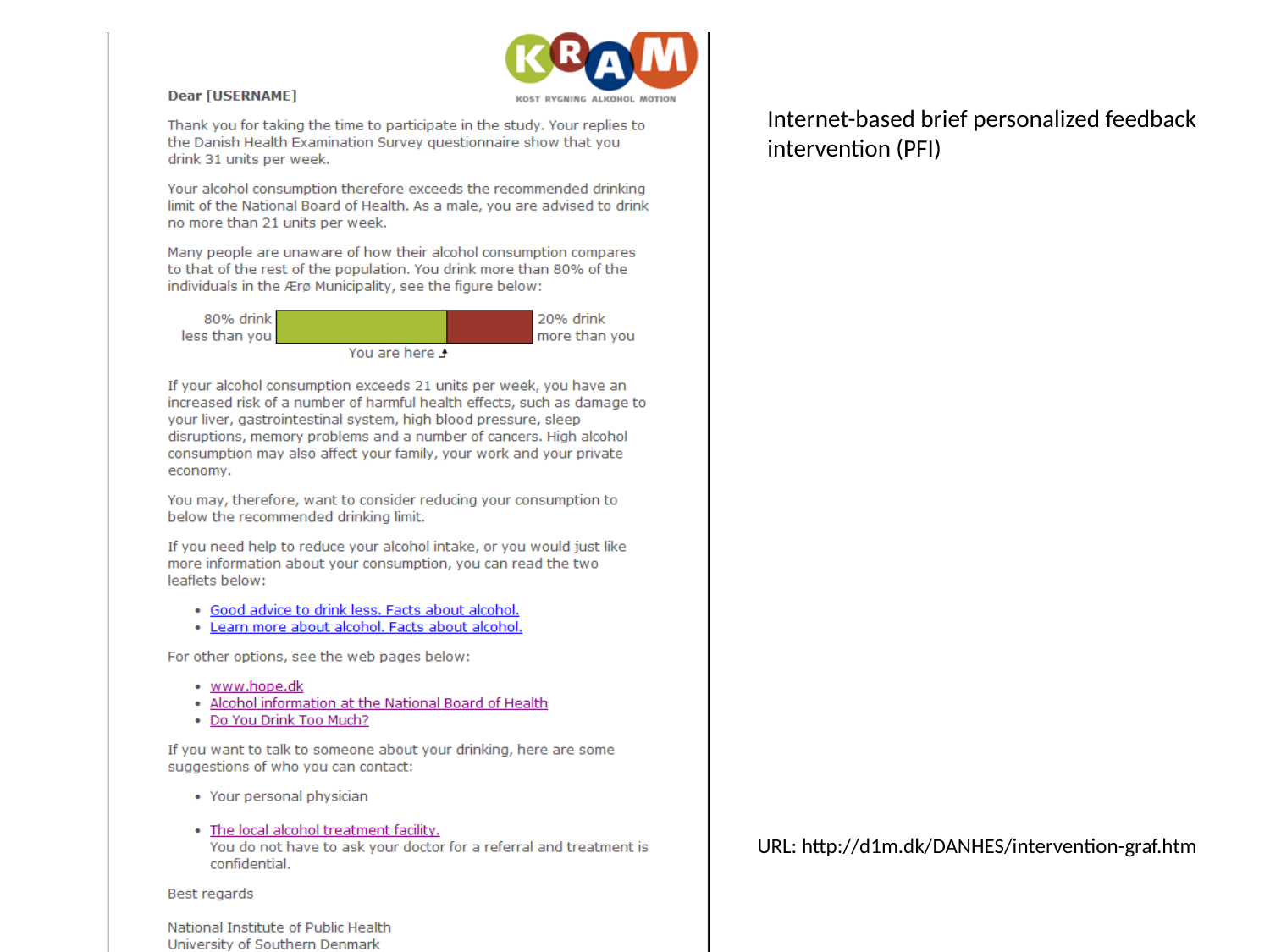

Internet-based brief personalized feedback intervention (PFI)
URL: http://d1m.dk/DANHES/intervention-graf.htm

## Slide 6
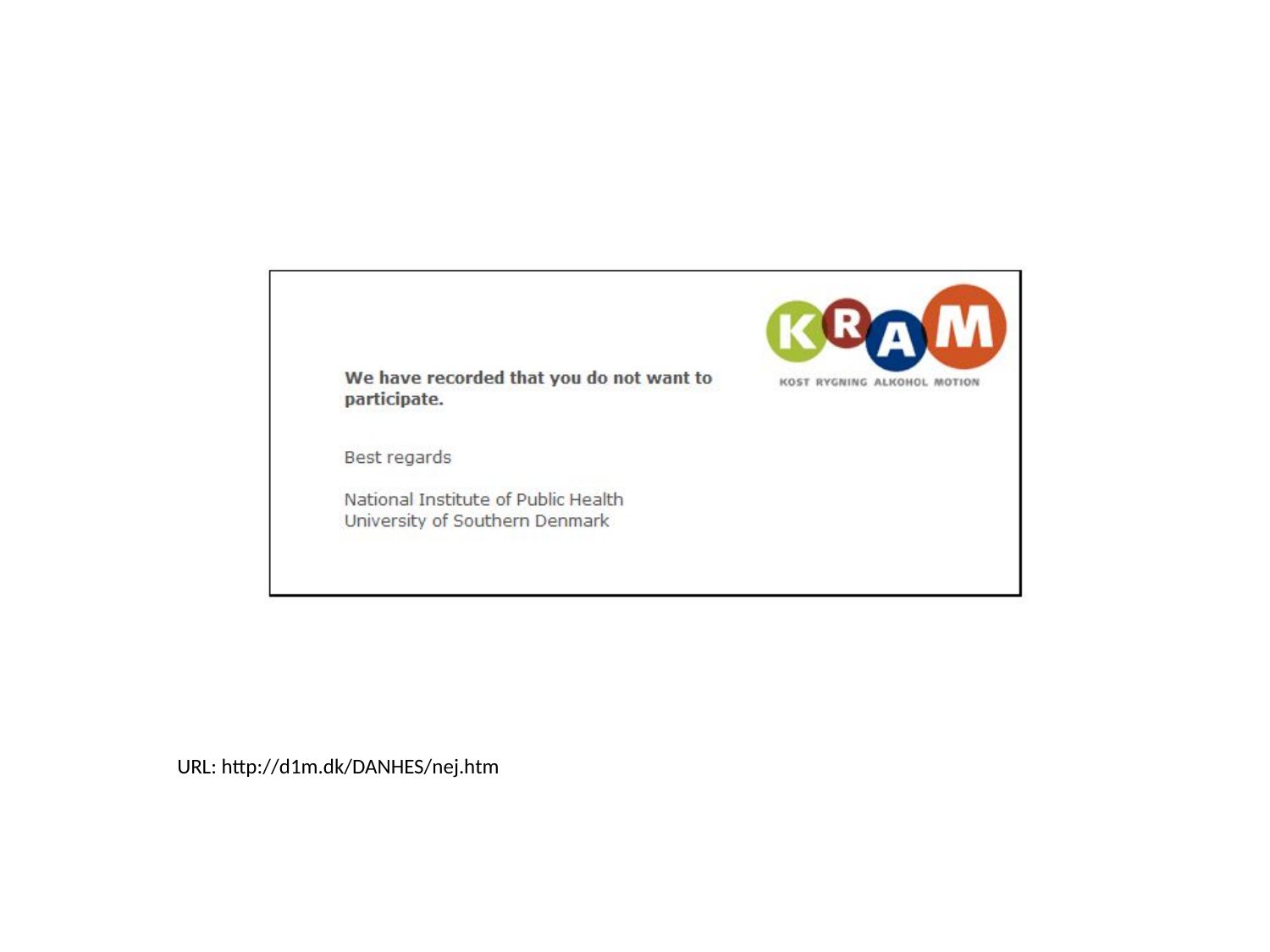

URL: http://d1m.dk/DANHES/nej.htm

## Slide 7
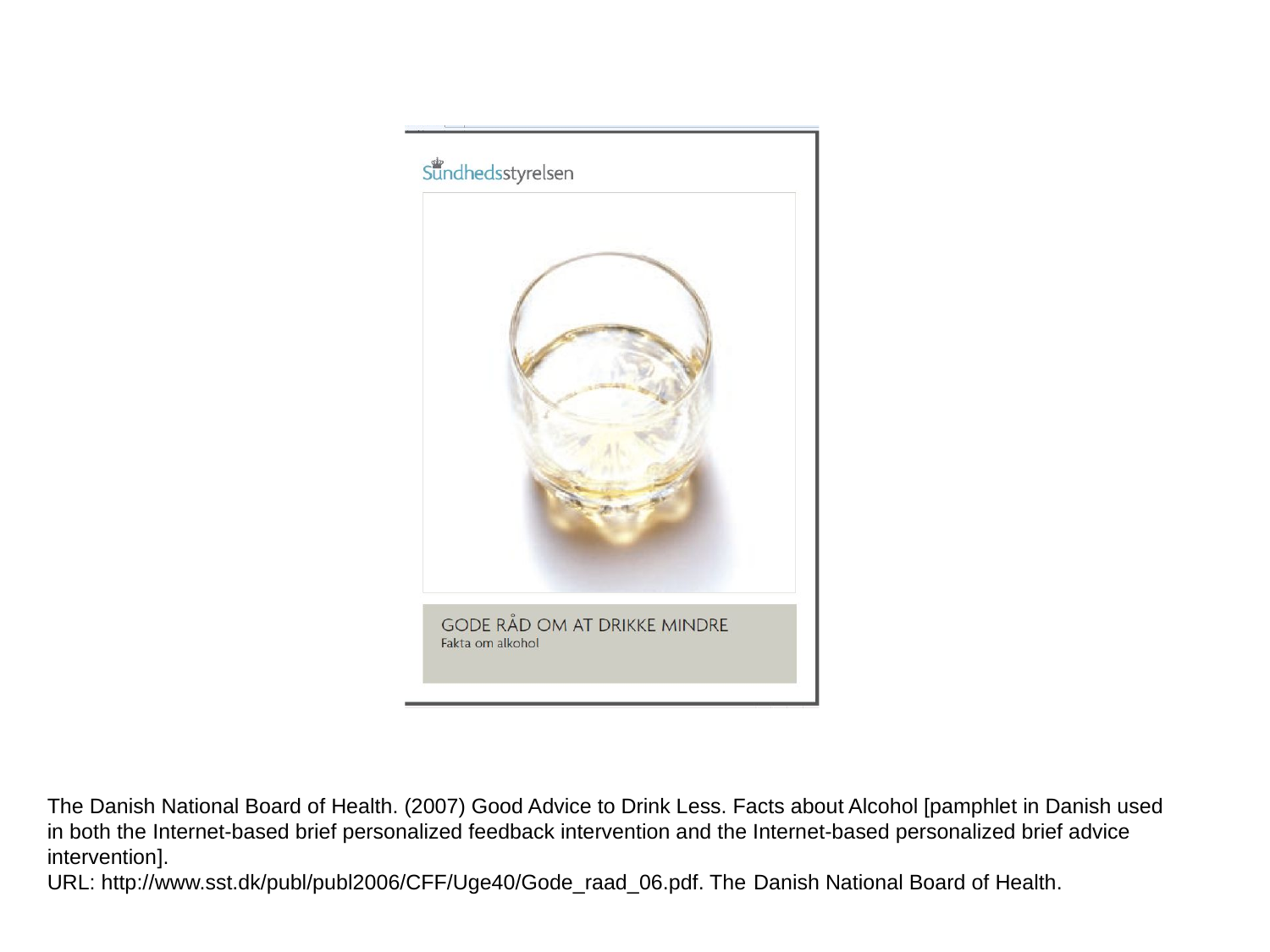

The Danish National Board of Health. (2007) Good Advice to Drink Less. Facts about Alcohol [pamphlet in Danish used in both the Internet-based brief personalized feedback intervention and the Internet-based personalized brief advice intervention].
URL: http://www.sst.dk/publ/publ2006/CFF/Uge40/Gode_raad_06.pdf. The Danish National Board of Health.

## Slide 8
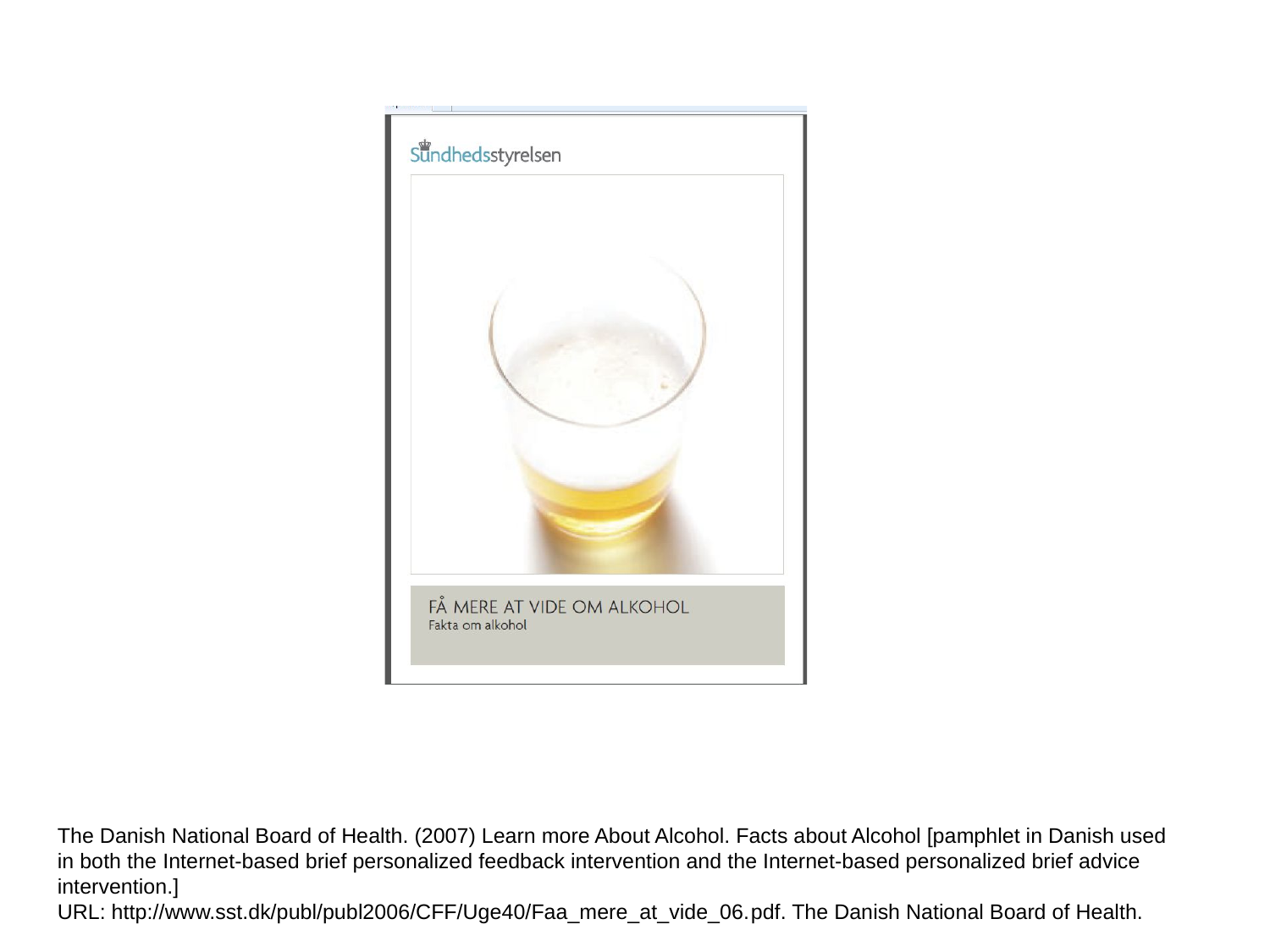

The Danish National Board of Health. (2007) Learn more About Alcohol. Facts about Alcohol [pamphlet in Danish used in both the Internet-based brief personalized feedback intervention and the Internet-based personalized brief advice intervention.]
URL: http://www.sst.dk/publ/publ2006/CFF/Uge40/Faa_mere_at_vide_06.pdf. The Danish National Board of Health.

## Slide 9
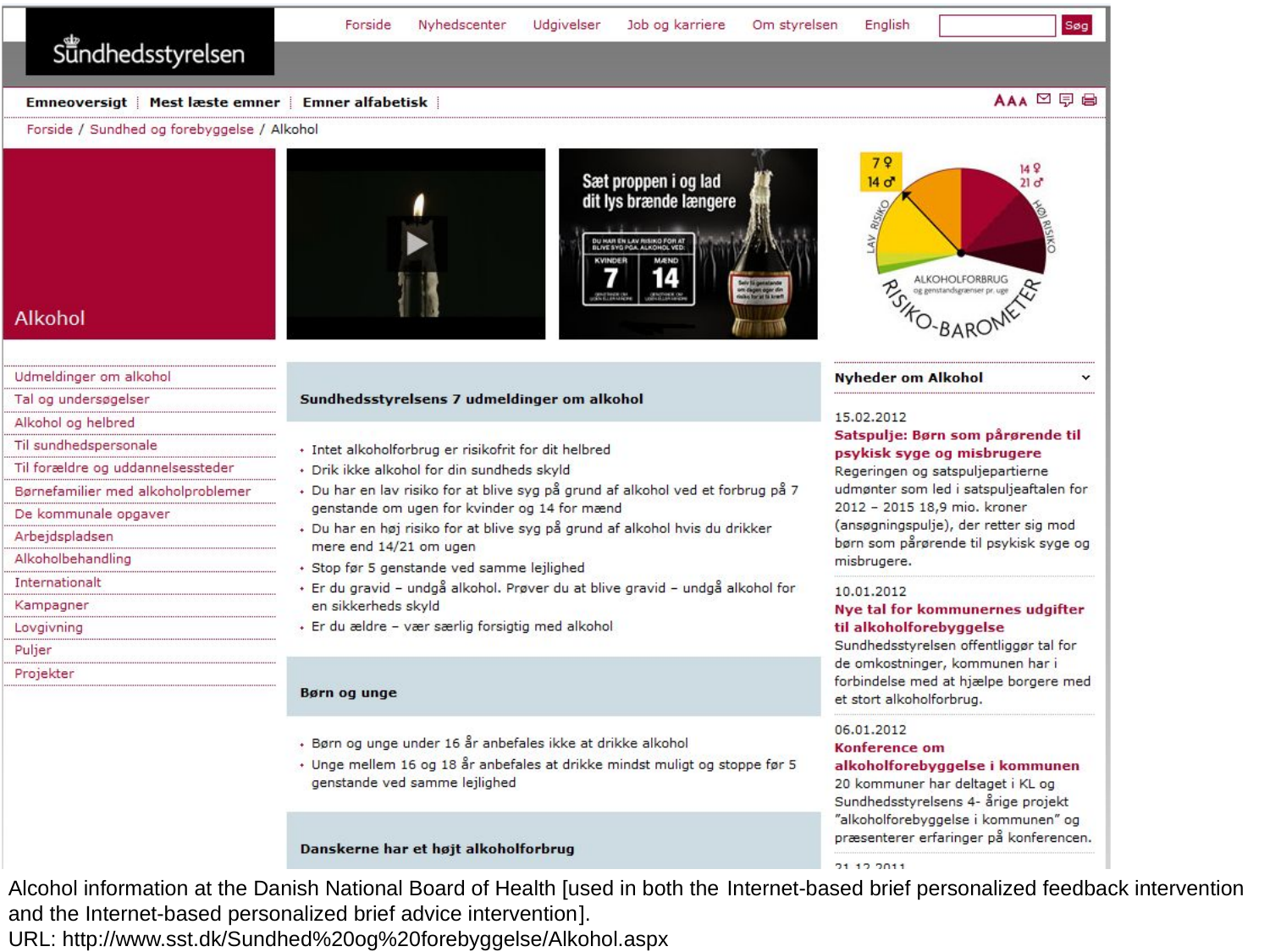

Alcohol information at the Danish National Board of Health [used in both the Internet-based brief personalized feedback intervention and the Internet-based personalized brief advice intervention].
URL: http://www.sst.dk/Sundhed%20og%20forebyggelse/Alkohol.aspx

## Slide 10
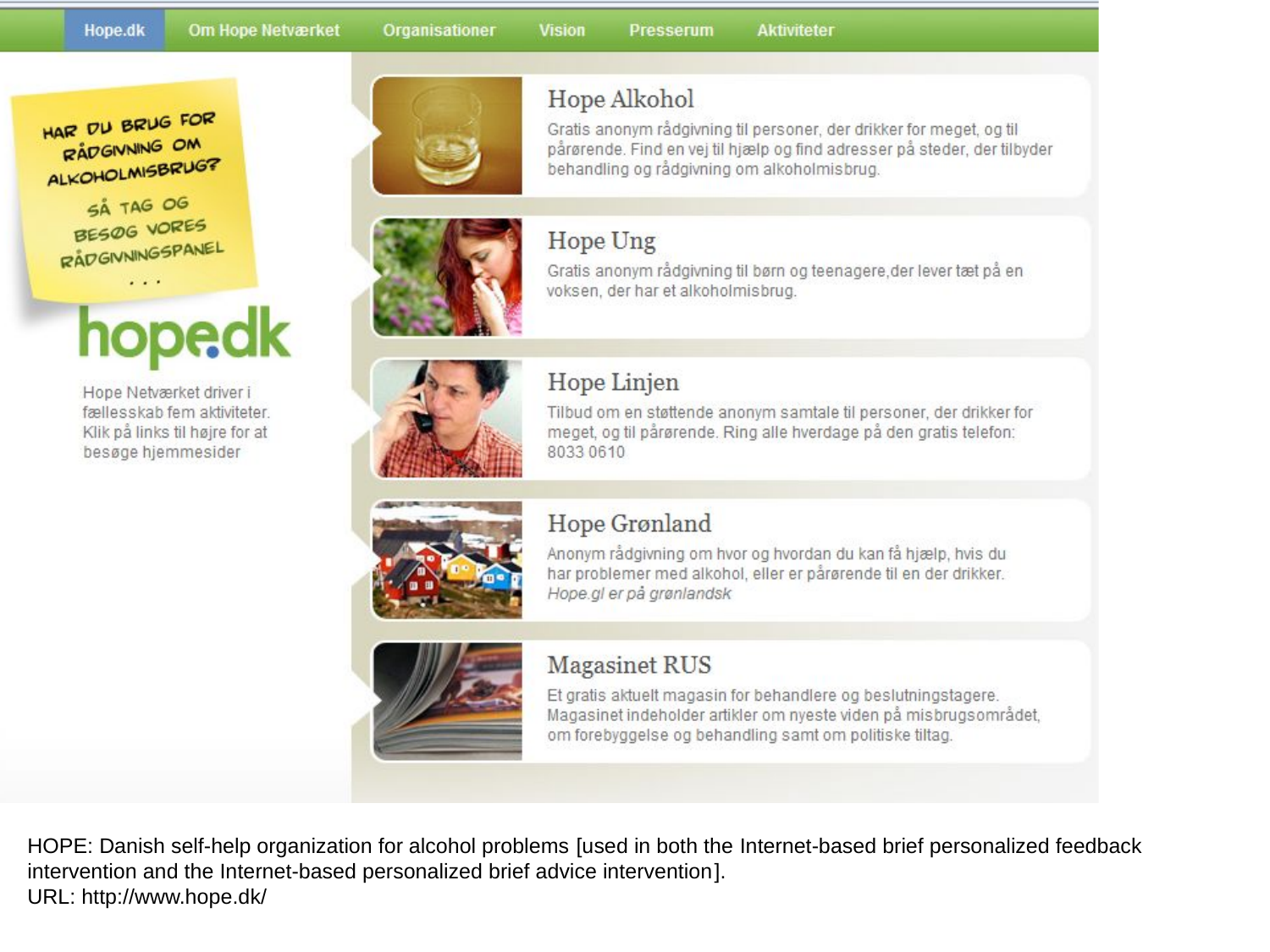

HOPE: Danish self-help organization for alcohol problems [used in both the Internet-based brief personalized feedback intervention and the Internet-based personalized brief advice intervention].
URL: http://www.hope.dk/

## Slide 11
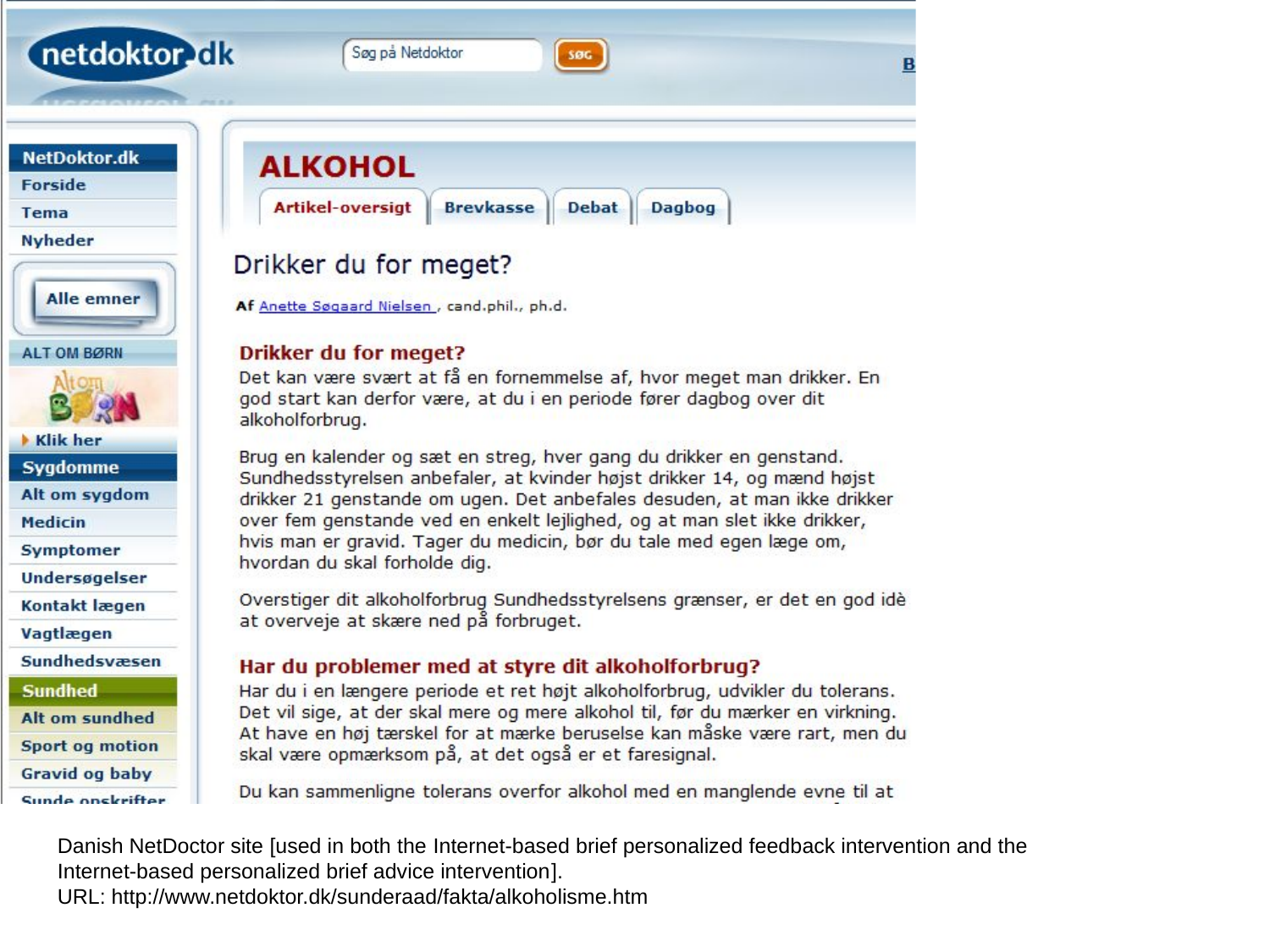

Danish NetDoctor site [used in both the Internet-based brief personalized feedback intervention and the Internet-based personalized brief advice intervention].
URL: http://www.netdoktor.dk/sunderaad/fakta/alkoholisme.htm
