# Supplementary material for: Internet-Based Brief Personalized Feedback Intervention in a Non-Treatment-Seeking Population of Adult Heavy Drinkers: A Randomized Controlled Trial
Source: J Med Internet Res. 2012 Jul 30;14(4):e98. doi: 10.2196/jmir.1883 (PMC3409578; doi:10.2196/jmir.1883)
Supplement: Supplementary file 2 [file jmir_v14i4e98_app2.pdf]

CONSORT-EHEALTH Checklist V1.6 Report	Manuscript Number	1883
Date completed	8/25/2011 14:50:29	
by	Anders Blædel Gottlieb Hansen	
Internet-based brief personalized feedback intervention in a Non-Treatment Seeking Population of Adult Heavy Drinkers: a randomized controlled trial		
TITLE		
**1a-i) Identify the mode of delivery in the title**		
Yes, see below:<br>"Internet-based brief personalized feedback intervention in a Non-Treatment Seeking Population of Adult Heavy Drinkers: a randomized controlled trial"		
**1a-ii) Non-web-based components or important co-interventions in title**		
Not relevant		
**1a-iii) Primary condition or target group in the title**		
Yes, the word "adult" heavy drinkers has been added to the title, see below:<br><br>"Internet-based brief personalized feedback intervention in a Non-Treatment Seeking Population of Adult Heavy Drinkers: a randomized controlled trial"		
ABSTRACT		
**1b-i) Key features/functionalities/components of the intervention and comparator in the METHODS section of the ABSTRACT**		
Yes, see abstract below:<br><br>"Objective:<br>To test if an Internet-based brief personalized feedback intervention (PFI) and an Internet-based personalized brief advice intervention (PBA) in a non-treatment seeking population of heavy drinkers result in a reduced alcohol intake.<br>Methods:<br>We conducted a three-arm parallel randomized controlled trial in a general population-based sample of heavy drinkers. The 54,157 participants (median age of 58 years) were screened for heavy drinking. Of 3418 participants who had a weekly alcohol consumption above 14 drinks for women and 21 drinks for men, 1380 (619 women) consented to the trial and were randomized to receive a PFI (normative feedback, n=476), a group receiving PBA (n=450) or a non-intervention control group (n=454). Follow-up took place after 6 and 12 months including 871 and 1064 participants respectively. The outcome measure was self-reported weekly alcohol consumption. Data were analyzed according to intention-to-treat principle and to examine changes over time and to account for the multiple time measurements we used a multilevel linear mixed model. To take attrition into account we used multiple imputation to address missing data."		
**1b-ii) Level of human involvement in the METHODS section of the ABSTRACT**		
Yes, this information has been added in the abstract and in the methods section, see below:<br><br>"To test if a fully automated Internet-based brief personalized feedback intervention (PFI) and a fully automated Internet-based personalized brief advice intervention (PBA) in a non-treatment seeking population of heavy drinkers result in a reduced alcohol intake."<br><br>"The PFI was a fully automated single-session intervention"<br><br>"The PBA was a fully automated single-session intervention and was displayed in a single screenshot and addressed the participant by name"		
**1b-iii) Open vs. closed, web-based (self-assessment) vs. face-to-face assessments in the METHODS section of the ABSTRACT**		
Yes, see below:<br><br>"Outcome measure and follow-up<br>The outcome measure was specified a priori and was based on self-reported drinking each day in a typical week and included beverage-specific questions (beer, wine, fortified wine, and spirits). Follow-up at 6 and 12 month contained the same alcohol items included in the baseline questionnaire and was conducted using an Internet-based questionnaire that participants accessed using a link provided in an e-mail. Follow-up at 12 month was furthermore supplemented by a letter containing the questionnaire, if the participants did not respond to the e-mail."		
**1b-iv) RESULTS section in abstract must contain use data**		
Yes, see below. However information on attrition appear in the "Methods" in the abstract<br><br>"Follow-up took place after 6 and 12 months including 871 and 1064 participants respectively"		
**1b-v) CONCLUSIONS/DISCUSSION in abstract for negative trials**		
Yes, we do take this into account, see below:<br><br>"Conclusions:<br>In this randomized controlled trial we found indications that an Internet-based PFI could be effective in reducing drinking in an adult population of heavy drinkers. Due to our design with two intervention groups and a pure control group, this study provides preliminary evidence that the active component in our interventions is feedback of one's own drinking relative to normative standards."		
INTRODUCTION		
**2a-i) Problem and the type of system/solution**		
Yes, see below:<br>"Drawbacks to the previous personalized feedback intervention studies are small sample sizes, short-term follow-up, few studies in non-student adult populations, few comparisons with appropriate control groups and high rates of attrition [14,16,23]. In the present study we address these shortcomings by comparing an Internet-based single-session PFI with an Internet-based PBA against a pure control group in the context of a Danish Health Examination Survey (DANHES) [24]. We seek to determine whether a single session PFI and PBA, resulted in lowering of alcohol use in a non-treatment seeking population of adult heavy drinkers. As secondary aims, we also wished to determine if any gender specific effects of the PFI could be found, especially because ambiguity remains when it comes to differential effectiveness between genders in non-internet brief interventions [4,25,26] and because few IBI studies presents data separately for men and women."		
**2a-ii) Scientific background, rationale: What is known about the (type of) system**		
Yes, see below:<br><br>"Unresolved questions exists, such as the need to establish what components of IBI that are effective [18]. Providing a personalized feedback intervention that compares one's own drinking and peers' actual drinking, have been found to enhance motivation to change drinking by making individuals aware of discrepancies between current alcohol use and social norms. Originating from self-regulation theory, this approach builds on the assumption that change is triggered by creating an awareness of a perceived discrepancy and if heavy drinkers finds no such they would view their own behaviour as normative rather than abnormal. This personal tailored approach, also termed normative feedback, as it tries to create behaviour change by targeting normative misperceptions is assumed to be more effective in reducing drinking than delivering standardized feedback in the form of self-help material [16,19-22]."		
METHODS		
**3a) CONSORT**		
Yes, see below:<br><br>"In the present study we address these shortcomings by comparing an Internet-based single-session PFI with an Internet-based PBA against a pure control group in the context of a Danish Health Examination Survey (DANHES) [24]. We seek to determine whether a single session PFI and PBA, resulted in lowering of alcohol use in a non-treatment seeking population of adult heavy drinkers. As secondary aims, we also wished to determine if any gender specific effects of the PFI could be found, especially because ambiguity remains when it comes to differential effectiveness between genders in non-internet brief interventions [4,25,26] and because few IBI studies presents data separately for men and women."		
**3b-i) Bug fixes, Downtimes, Content Changes**		
not relevant		
**4a-i) Computer / Internet literacy**		

Yes, see below (from discussion):

"Due to our recruitment of participants from a sample where almost everyone had internet access, Internet- and computer literacy was low in our sample. When generalizing to the Danish population, it should be borne in mind, that 86% of the Danes have Internet access."

4a-ii) Open vs. closed, web-based vs. face-to-face assessments:

Yes, see below (from "Methods"):

"Procedure
After completing the Internet-based DANHES questionnaire, invitees were automated screened and heavy drinkers identified. Heavy drinkers received an e-mail inviting them to participate in the intervention study. By clicking on a link in the e-mail invitees were directed to a secure website where they, after entering user name and personal access code (provided in DANHES invitation letter), were directed to another website explaining them about the study (see Multimedia Appendix). After providing online consent, participants were automatically randomized and directed to a new personalized website presenting them to the interventions or control, which were displayed immediately on the screen."

4a-iii) Information giving during recruitment

Yes, see Multimedia appendix

4b-i) Report if outcomes were (self-)assessed through online questionnaires

Yes, see below (from "Methods"):

"Outcome measure and follow-up
The outcome measure was specified a priori and was based on self-reported drinking each day in a typical week and included beverage-specific questions (beer, wine, fortified wine, and spirits). Follow-up at 6 and 12 month contained the same alcohol items included in the baseline questionnaire and was conducted using an Internet-based questionnaire that participants accessed using a link provided in an e-mail. Follow-up at 12 month was furthermore supplemented by a letter containing the questionnaire, if the participants did not respond to the e-mail."

4b-ii) Report how institutional affiliations are displayed

Yes, see Multimedia Appendix

5-i) Mention names, credential, affiliations of the developers, sponsors, and owners

Not relevant, however see below:

"Acknowledgements
No conflict of interests.
The study was approved by the Danish local ethics committee and the Danish Data Protection Agency and all local confidentiality and privacy requirements have been met.
The study was funded by the National Board of Health, Denmark. The funder had no role in study design, data collection and analysis, decision to publish, or preparation of the manuscript."

5-ii) Describe the history/development process

Not relevant

5-iii) Revisions and updating

Not relevant

5-iv) Quality assurance methods

Not applicable

5-v) Ensure replicability by publishing the source code, and/or providing screenshots/screen-capture video, and/or providing flowcharts of the algorithms used

Yes, see Multimedia appendix

5-vi) Digital preservation

Yes, see Multimedia appendix

5-vii) Access

Yes, see below (from "Methods"):

"Procedure
After completing the Internet-based DANHES questionnaire, invitees were automated screened and heavy drinkers identified. Heavy drinkers received an e-mail inviting them to participate in the intervention study. By clicking on a link in the e-mail invitees were directed to a secure website where they, after entering user name and personal access code (provided in DANHES invitation letter), were directed to another website explaining them about the study (see Multimedia Appendix). After providing online consent, participants were automatically randomized and directed to a new personalized website presenting them to the interventions or control, which were displayed immediately on the screen."

5-viii) Mode of delivery, features/functionalities/components of the intervention and comparator, and the theoretical framework

Yes, see below (from "Methods"):

"Interventions
The PFI was a fully automated single-session intervention; it was displayed in a single screenshot and addressed the participant by name. It consisted of a summary of the participant's weekly consumption, a comparison with maximum drinking limits and a graphical comparison of the participant's consumption to the average level in the municipality (gender-specific), followed by information about health and social risks of heavy drinking as well as links for further self-help material and a local alcohol treatment facility (see Multimedia Appendix).
The PBA was a fully automated single-session intervention and was displayed in a single screenshot and addressed the participant by name. It informed the participant that his/hers alcohol consumption exceeded the recommended maximum drinking limits, followed by information about health and social risks of heavy drinking as well as links for further standardized self-help material and a local alcohol treatment facility (see Multimedia Appendix).What distinguish the PFI from the PBA, is the normative component and the summary of the participant's weekly alcohol consumption; common for the two interventions is the information on adverse effects of heavy drinking, advice to cut down and links for further material.
Participants in the control group received a single screenshot explaining them that being randomized to the control group implied no intervention and follow-up after 6- and 12 months (see Multimedia Appendix).
Persons below the maximum drinking limits did not receive any feedback or interventions."

5-ix) Describe use parameters

Not relevant, however see Multimedia appendix.

5-x) Clarify the level of human involvement

Not relevant (interventions were fully automated).

5-xi) Report any prompts/reminders used

Not relevant

5-xii) Describe any co-interventions (incl. training/support)

Not relevant

6a-i) Online questionnaires: describe if they were validated for online use and apply CHERRIES items to describe how the questionnaires were designed/deployed

Yes, but i have no knowledge of online validation of the questionaire we used, but see below:

"The alcohol questions were beverage-specific (beer, wine, fortified wine, spirits) and asked for consumption each day in a typical week. Additionally the Alcohol Use Disorder Identification Test (AUDIT) questions 3 – 10 were included (timeframe: preceding 12 months)."

We have these reflections in the discussion:

"We relied on measuring outcome using self-reports of alcohol consumption, which is a method that has demonstrated reasonable levels of accuracy [37,43] and we have tried to minimize the bias of underreporting by asking beverage-specific questions, which have been shown to yield higher volumes than questions only for total alcohol consumption [44]."

6a-ii) Describe whether and how "use" (including intensity of use/dosage) was defined/measured/monitored

not relevant

6a-iii) Describe whether, how, and when qualitative feedback from participants was obtained

not relevant

7a-i) Describe whether and how expected attrition was taken into account when calculating the sample size

Yes, see below:

"Power estimates
Reduction in past week alcohol consumption can be expected to be 15% - 20% for the PFI group, 5% - 10% for the PBA group and 5% - 10% for the control group [30,31]. Making these assumptions led to the calculation that approximately 400 participants providing follow-up data would be required to give 80% power at the 5% significance level to detect a 20% vs. 10% difference in the past week's reported alcohol consumption between intervention and control groups."

7b) CONSORT

not relevant

8a) CONSORT

Yes, see below:

"Randomization
Eligible persons were randomly assigned and enrolled into PFI, PBA or control group by the web server software implemented by a technician who was not involved in the recruitment process. Blinding was not feasible. Participants did not know which of the two interventions was the 'intervention of interest'. Prior to randomization all three groups were informed about the purpose of the study and the nature of the control group (see Multimedia Appendix)."

8b) CONSORT

Yes, see above.

9) CONSORT

Yes, see above.

10) CONSORT

Yes, see above.

11a-i) Specify who was blinded, and who wasn't

Yes, see below:

"Randomization
Eligible persons were randomly assigned and enrolled into PFI, PBA or control group by the web server software implemented by a technician who was not involved in the recruitment process. Blinding was not feasible. Participants did not know which of the two interventions was the 'intervention of interest'. Prior to randomization all three groups were informed about the purpose of the study and the nature of the control group (see Multimedia Appendix)."

11a-ii) Discuss e.g., whether participants knew which intervention was the "intervention of interest" and which one was the "comparator"

Yes, see below:

"Randomization
Eligible persons were randomly assigned and enrolled into PFI, PBA or control group by the web server software implemented by a technician who was not involved in the recruitment process. Blinding was not feasible. Participants did not know which of the two interventions was the 'intervention of interest'. Prior to randomization all three groups were informed about the purpose of the study and the nature of the control group (see Multimedia Appendix)."

11b) CONSORT

not relevant

12a) CONSORT

Yes, see below:

"Statistical analysis
The primary and secondary analyses are based on the intention-to-treat principle (ITT) and concern the mean difference in changes in alcohol consumption between the two intervention groups and control group.
Analyses were carried out using Stata version 11.2 (StataCorp, USA). Quantitative variables were described by the mean and standard deviation (SD), by the median and its interquartile range (IQR) or by its 95% confidence interval (95% CI). In all tests, $p < .05$ was chosen as the level of significance.
The residuals were approximately normally distributed. Hence, to examine changes over time and to account for the multiple time measurements data were analyzed by using a multilevel mixed model, using the xtmixed procedure. The model examined fixed effects for alcohol consumption, group, sex and month and a random intercept to account for clustering within each participant. The model also included an interaction term between intervention group and month, allowing differences in the intervention effect between follow-up assessments [32]. The fixed effect of most interest was the month-group interaction effect that indicated the difference between intervention groups and control group in change over time of alcohol consumption.
For comparison of the three groups in the secondary analyses, the Kruskal-Wallis non-parametric test for continuous variables was used.
For the loss to follow-up analysis a logistic regression model with participation in follow-up as the dependent variable was used (for explanatory variables see table 1). In a pre-planned analysis we used multiple imputation to take attrition into account for participants who did not complete the 6- or 12-month follow-up. Multiple imputation allows for the uncertainty about the missing data by creating several different plausible imputed data sets and appropriately combining results obtained from each of them (we generated 20 data sets), which will often provide a more reliable approach than complete-case analysis in the presence of missing data [23,33]. For this we used the mi impute mvn procedure, which uses multivariate normal regression for continuous data and assumes that data are missing at random [34]. As sensitivity analyses we also report results from: (1) an analysis of all available results without imputation of missing data (completers only analysis) and (2) an analysis with simple imputation (last observation carried forward) assuming that non-responders have no change in their alcohol consumption."

12a-i) Imputation techniques to deal with attrition / missing values

Yes, see above.

12b) CONSORT

see above.

RESULTS

13a) CONSORT

See flow chart (figure 1) and below:

"Participant Flow
Of the 54,157 persons screened, 3418 were heavy drinkers (6%). Of these, 785 refused participation (23%), and 1215 did not respond to the invitation mail (36%). In total 1380 persons accepted participation (40%) and were randomly assigned into PFI (n=476), PBA (n=450) or control group (n=454). The 6 month follow-up was completed by 871 persons (63% of enrolled participants) and the 12 month follow-up was completed by 1064 persons (77%) (Figure 1)."

13b) CONSORT

See flow chart (figure 1).

13b-i) Attrition diagram

This item is described in the text and the information does also appear in the flow char (figure 1). see below:

"Participant Flow
Of the 54,157 persons screened, 3418 were heavy drinkers (6%). Of these, 785 refused participation (23%), and 1215 did not respond to the invitation mail (36%). In total 1380 persons accepted participation (40%) and were randomly assigned into PFI (n=476), SHMPBA (n=450) or control group (n=454). The 6 month follow-up was completed by 871 persons (63% of enrolled participants) and the 12 month follow-up was completed by 1064 persons (77%) (Figure 1)."

14a) CONSORT

Yes, see below:

"Recruitment
Recruitment for the study began September 2008. Follow-up started February 2009 and ended in February 2010."

14a-i) Indicate if critical “secular events” fell into the study period

not relevant

14b) CONSORT

not relevant

15) CONSORT

Yes, see table 1.

15-i) Report demographics associated with digital divide issues

Not known.

16-i) Report multiple “denominators” and provide definitions

Yes, we did ITT-analysis, se methods and flow chart (figure 1).

"The primary and secondary analyses are based on the intention-to-treat principle (ITT) and concern the mean difference in changes in alcohol consumption between the two intervention groups and control group."

16-ii) Primary analysis should be intent-to-treat

Yes, se above and below:

"As sensitivity analyses we also report results from: (1) an analysis of all available results without imputation of missing data (completers only analysis) and (2) an analysis with simple imputation (last observation carried forward) assuming that non-responders have no change in their alcohol consumption."

17a) CONSORT

Yes, se results and Table 2:

"Outcomes
Descriptive statistics for alcohol consumption, by gender and group, at baseline, 6- and 12 month follow-up using multipel imputation, are shown in figure 2a and 2b. Consumption among women decreased from a mean baseline level of 21.0 drinks/week to 16.7 drinks/week for the control group (95% CI: 14.7 to 18.8), 16.0 drinks/week for the PFI (95% CI: 14.2 to 17.9) and 17.0 for the SHMPBA (95% CI: 14.6 to 19.5) after 6 months (Figure 2a). Consumption among men decreased from a mean baseline level of 32.0 drinks/week to 26.7 drinks/week for the control group (95% CI: 25.0 to 28.4), 25.1 drinks/week for the PFI (95% CI: 23.2 to 27.1) and 26.9 drinks/week for the SHM PBA (95% CI: 24.9 to 28.8) after 6 months (Figure 2b). Figures for 12 month follow-up were approximately similar (Figure 2a and 2b)."

17a-i) Presentation of process outcomes such as metrics of use and intensity of use

not relevant

17b) CONSORT

not relevant

18) CONSORT

not relevant

18-i) Subgroup analysis of comparing only users

Yes is stressed in the discussion, see below:

"However, it must be stressed that when interpreting the results from the completers analysis, we are dealing with a self-selected sample and no longer an unbiased sample from a randomized trial, and hence no clear conclusions regarding intervention efficacy can be drawn."

19) CONSORT

not relevant

19-i) Include privacy breaches, technical problems

not relevant

19-ii) Include qualitative feedback from participants or observations from staff/researchers

not relevant

DISCUSSION

20-i) Typical limitations in ehealth trials

Yes, see below:

"Attrition (37% at 6 months and 23% at 12 months) is an area of concern in our study, as it could introduce a selection bias, causing imbalance among the previously randomized groups and thus, be a threat to internal validity. This was partly confirmed by our analysis, which revealed differential attrition. Participants lost to follow-up from the PFI group were more likely not to be motivated to cut down on their drinking and more likely to be binge drinkers and heavy smokers. Participants in the control group lost to follow-up, were more likely to consume more than 49 drinks/week and more likely to be heavy smokers, whereas participants in the PBA group were less likely to be aged between 45 and 65 years. The importance of dealing with this complicated picture of differential attrition is underlined by the sensitivity analyses, which reveal that an analysis of completers only or an analysis using ‘last observation carried forward’ will more than likely lead to an overestimation of treatment effects. By using multipel imputation in our main analysis we have provided a plausible estimate of what might have been shown if no attrition had occurred. Generalizability of the present findings is restricted due to the under-representation of individuals with the lowest level of education, unmarried individuals, and younger persons in our population. However, generalizability is a general problem of much brief intervention research dealing with populations which are not representative of the population of heavy drinkers [37,42]. This was confirmed by our results, which showed that in the DANHES population, 6% were heavy drinkers, compared to a 20% prevalence estimated for the Danish population [3]. Due to our recruitment of participants from a sample where almost everyone had internet access, Internet- and computer literacy was low in our sample. When generalizing to the Danish population, it should be borne in mind, that 86% of the Danes have Internet access. The results must also be interpreted in consideration of the fact that the use of a health examination survey to proactively enlist heavy drinkers (who were not seeking help) may have resulted in a preponderance of heavy drinkers with low levels of alcohol-related harm.
We relied on measuring outcome using self-reports of alcohol consumption, which is a method that has demonstrated reasonable levels of accuracy [37,43] and we have tried to minimize the bias of underreporting by asking beverage-specific questions, which have been shown to yield higher volumes than questions only for total alcohol consumption [44].
"

21-i) Generalizability to other populations

Yes, see above.

21-ii) Discuss if there were elements in the RCT that would be different in a routine application setting

I many ways our trial resemble an effectiveness trial. See below:

"In this rigorously conducted trial we succeeded in implementing an IBI in a general population-based sample of heavy drinkers. The naturalistic setting of the trial (i.e. the participant accessed the intervention in their own home) increases confidence regarding the generalizability of the results [18,39]. Another strength of a naturalistic trial design is that it elucidates important feasibility aspects of reaching a non-treatment seeking population of heavy drinkers by e-mail. Knowing that 36% of the invitees did not respond to the invitation mail and that 23% refused participation, is applicable knowledge when designing and disseminating similar interventions.
Our aim was to investigate how minimal an IBI can be while still retaining an impact on drinking. Thus the interventions were displayed in a single screenshot immediately after providing online consent, and we avoided the problem of knowing whether the participants randomized to the interventions actually used the interventions [39]."

22-i) Restate study questions and summarize the answers suggested by the data, starting with primary outcomes and process outcomes (use)

Yes, see below:<br><br>"Discussion<br><br>Key findings<br>In this randomized controlled trial we found indications that an Internet-based brief personalized feedback intervention to could be effective in reducing drinking in an adult population of heavy drinkers. The intervention effect of the PFI was approximately 2 drinks/week and of approaching borderline significance, but highly significant in the sensitivity analyses with an intervention effect of approximately 3 drinks/week for the PFI. In a secondary completers analysis of men, we found a significant difference of 3.5 drinks/week between the PFI and the control group at 6 month follow-up. However, it must be stressed that when interpreting the results from the completers analysis, we are dealing with a self-selected sample and no longer an unbiased sample from a randomized trial, and hence no clear conclusions regarding intervention efficacy can be drawn. From baseline to 6- and 12 month follow-up, alcohol consumption declined significantly in both intervention groups and the control group with approximately 6 drinks per week."		
**22-ii) Highlight unanswered new questions, suggest future research**		
Yes, see below:<br><br>"Due to our design with two intervention groups and a pure control group, this study provides preliminary evidence that the active component in our interventions is feedback of one's own drinking relative to normative standards. This knowledge can be utilized in the design of future alcohol interventions. Knowing the mechanism of change is an important contribution to the IBI field, as the existing research in this area has been focused on college samples [41]"		
Other information		
**23) CONSORT**		
ClinicalTrials.gov registration NCT00751985.		
**24) CONSORT**		
full protocol can not be accessed (is only in Danish), however se above.		
**25) CONSORT**		
Yes, se below:<br><br>"The study was funded by the National Board of Health, Denmark. The funder had no role in study design, data collection and analysis, decision to publish, or preparation of the manuscript."		
**X26-i) Comment on ethics committee approval**		
Yes, se below:<br><br>"The study was approved by the Danish local ethics committee and the Danish Data Protection Agency and all local confidentiality and privacy requirements have been met."		
**x26-ii) Outline informed consent procedures**		
Yes, see Multimedia appendix		
**X26-iii) Safety and security procedures**		
We only used secure website (i.e. https://), see below:<br><br>"By clicking on a link in the e-mail invitees were directed to a secure website where they, after entering user name and personal identification numberaccess code (provided in DANHES invitation letter), were directed to another website explaining them about the study (see Multimedia Appendix)."		
**X27-i) State the relation of the study team towards the system being evaluated**		
Yes, se below:<br><br>"Acknowledgements<br>No conflict of interests."		
